# LARP4A recognizes polyA RNA via a novel binding mechanism mediated by disordered regions and involving the PAM2w motif, revealing interplay between PABP, LARP4A and mRNA

**DOI:** 10.1093/nar/gkz144

**Published:** 2019-03-01

**Authors:** Isabel Cruz-Gallardo, Luigi Martino, Geoff Kelly, R Andrew Atkinson, Roberta Trotta, Stefano De Tito, Pierre Coleman, Zainab Ahdash, Yifei Gu, Tam T T Bui, Maria R Conte

**Affiliations:** 1 Randall Centre for Cell and Molecular Biophysics, King’s College London, London SE1 1UL, UK; 2 MRC Biomedical NMR Centre, The Francis Crick Institute, London NW1 1AT, UK; 3 Centre for Biomolecular Spectroscopy, King’s College London, London SE1 1UL, UK; 4 Department of Chemistry, King’s College London, London SE1 1DB, UK

## Abstract

LARP4A belongs to the ancient RNA-binding protein superfamily of La-related proteins (LARPs). In humans, it acts mainly by stabilizing mRNAs, enhancing translation and controlling polyA lengths of heterologous mRNAs. These activities are known to implicate its association with mRNA, protein partners and translating ribosomes, albeit molecular details are missing. Here, we characterize the direct interaction between LARP4A, oligoA RNA and the MLLE domain of the PolyA-binding protein (PABP). Our study shows that LARP4A–oligoA association entails novel RNA recognition features involving the N-terminal region of the protein that exists in a semi-disordered state and lacks any recognizable RNA-binding motif. Against expectations, we show that the La module, the conserved RNA-binding unit across LARPs, is not the principal determinant for oligoA interaction, only contributing to binding to a limited degree. Furthermore, the variant PABP-interacting motif 2 (PAM2w) featured in the N-terminal region of LARP4A was found to be important for both RNA and PABP recognition, revealing a new role for this protein–protein binding motif. Our analysis demonstrates the mutual exclusive nature of the PAM2w-mediated interactions, thereby unveiling a tantalizing interplay between LARP4A, polyA and PABP.

## INTRODUCTION

LARP4A is a mainly cytoplasmic protein that promotes mRNA translation and stabilization, 3′ UTR polyA lengthening, post-transcriptional regulation of ribosomal protein production and miRNA processing (1–4). It interacts with poly(A), the PolyA-binding protein (PABP) and the receptor for activated C kinase (RACK1), and associates with translating polyribosomes (1). While a single LARP4 gene is found in invertebrate species, a gene duplication event very early in the vertebrate lineage gave rise to two variants termed LARP4A/LARP4 and LARP4B/LARP5 (5). We refer to these proteins as LARP4A and LARP4B henceforth. Although both proteins positively regulate protein synthesis, promote stability of a subset of mRNAs and share protein partners (PABP and RACK1) (1,6), they may have non-redundant functions regarding their RNA targets. LARP4A binds to oligoA sequences whereas LARP4B appears to prefer AU-rich regions (1,7), and recently LARP4A was identified as a regulator in microRNA mir-210 biogenesis (4). Both LARP4A and LARP4B appear to play key—and non-overlapping—roles in cancer. LARP4A controls cancer cell morphology and motility: gene depletion increases cell migration and invasion in prostate and breast cancer cells, whereas overexpression reduces cell elongation and favours cell circularity (8). LARP4B has been found to act as a tumour suppressor by a genetic screen in mice and human glioma cells (9,10).

LARP4A belongs to the La-related protein (LARP) superfamily, an ancient group of eukaryotic RNA-binding proteins (RBPs) whose importance in a myriad of cellular functions continues to emerge (2,5,11). LARPs share the distinctive RNA-binding locus called ‘La module’, composed of a La motif (LaM) paired with an RNA recognition motif (RRM1), which was first discovered in the La protein (2,12). The sequence similarities in La modules belie the fact different LARPs bind to very different RNA targets (2), and the molecular bases for such substrate discrimination remain a conundrum and a focus of investigations.

A high degree of sequence conservation is retained in LaMs of LARPs (2,5), whilst RRM1s vary across the families, albeit their exact contribution to specific RNA interaction remains elusive (2). By far, the best characterized La module belongs to the human La protein, which recognizes the short 3′UUU_OH_ tail of nascent RNA polymerase III transcripts and other non-coding RNAs, guarding them against the activity of 3′ exonucleases. Interaction of 3′UUU_OH_ with La places the nucleotide at the 3′ end inside a highly conserved pocket that is formed exclusively from LaM residues but is positioned close to the interface of the LaM and RRM1 domains. This terminal uridylate fits snugly into the LaM pocket, where it makes a bifurcated hydrogen bond with D33 and stacking interactions with F35 and F55. At the deepest recess of the binding cleft, the penultimate U makes extensive contacts with both LaM and RRM1, and the induced fit around this nucleotide accounts well for the cooperative nature of RNA binding by both domains of the La module (13–15). A network of specific La-UUU_OH_ contacts is established by six residues within the hydrophobic pocket of the LaM, namely Q20, Y23, Y24, D33, F35 and F55. Not only is this group of residues strikingly conserved across the superfamily, but also all actively participate in RNA interaction in other LARPs (16,17), in spite of the distinct RNA substrates recognized by other La modules (2). Only a subset of proteins within the LARP4 and LARP6 families do not possess an absolute conservation of these residues and, of relevance, LARP4A retains 4 out of the 6 conserved residues, being Y24 and F55 (human La numbering) substituted by C and M, respectively (11). Interestingly, the presence of this unconventional LaM correlates well with an evolutionary reorganization occurring in LARP4 and LARP6 families, led by the acquisition of a PAM2 motif (PABP-interacting motif 2) (11).

The PAM2 motif of LARP4A allows it to interact with PABP, in particular its C-terminal domain, denoted MLLE (1,18). Curiously, vertebrate LARP4 proteins contain an atypical PAM2 (dubbed PAM2w), in that a phenylalanine reported to be invariable and essential for PABP binding (at position ‘10’ of the consensus XX**L**XXX**A**XX**F**X**P** sequence) (18,19) is replaced by a tryptophan residue (11). Despite this amino acid substitution, the PAM2w appears functional and has remained fixed in all vertebrate LARP4A and LARP4B lineages underlying a strong selective pressure (1,6,11). In addition to the PAM2w motif, both LARP4A and LARP4B harbour a second binding site for PAPB, the PBM (PABP binding motif), located downstream of the RRM1 and for human LARP4A loosely charted to residues 287–358 (1). The C-terminal region of LARP4A and LARP4B also contains a RACK1-binding site (1,6).

**Figure 1.**
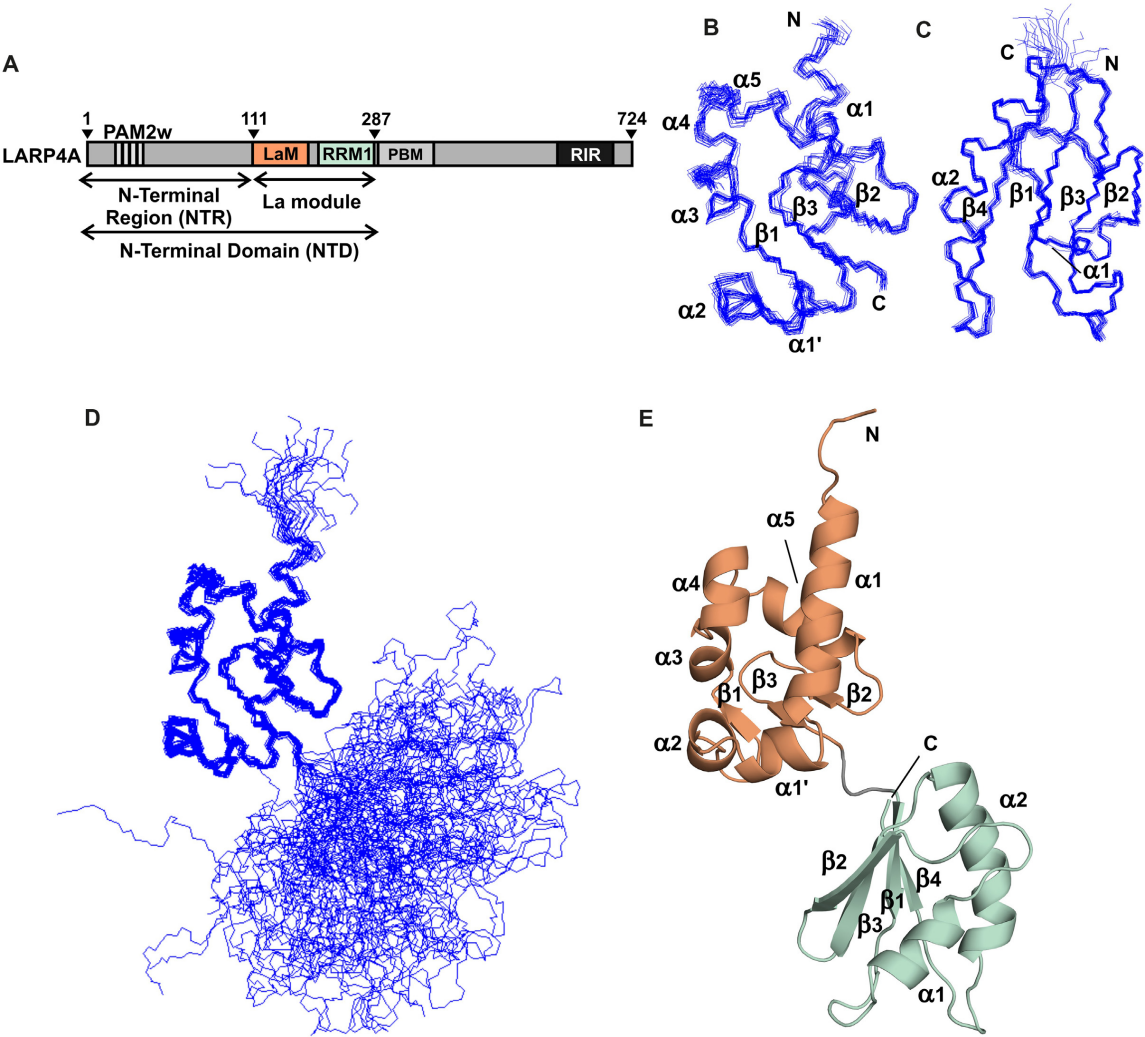
Domain organization of human LARP4A and solution structure of the La module. (**A**) Domain architecture of human LARP4A and nomenclature of fragments used in this study. The NTD spans residues 1–287 and the NTR residues 1–111. LARP4A La module, encompassing amino acids 111–287, is composed of two domains: the LaM (111–196) and the RRM1 (200–287). The PAM2w motif (13-26) and the PBM (287–358) are the PABP-binding sites, and RIR is the RACK1 interaction region. (**B–D**) Backbone traces of the 20 lowest energy structures of the La module, superposed separately on (B) the LaM (showing aa 118–196) and (C) the RRM1 (showing aa 199–275). (**D**) View of the entire La module superposed on LaM highlighting the non-fixed relative orientation of the two domains in solution. (**E**) Representative structure of LARP4A La module in cartoon representation. The LaM and the RRM1 are coloured as in panel (A). The N and C-termini, α-helices and β-strands are indicated.

The interaction of LARP4A with PABP has been suggested to be central for LARP4A functioning (2,3,20). PolyA-binding proteins are a major class of eukaryotic regulatory factors that associate with the 3′ polyA tail of mRNAs, with several roles in mediating gene expression. Cytoplasmic PABPs (PABPC), of which the best studied is PABPC1, contain four RRM domains connected to a C-terminal MLLE domain via a low complexity linker rich in proline and methionine residues (21,22). The RRMs perform oligoA interaction (21,23) and multiple PABP molecules can bind to the same polyA tract, forming a repeating unit of ∼27–30 nucleotides. Although RRMs of PABP can also mediate protein–protein contacts, its MLLE domain serves as the main protein-binding platform, associating with the many PAM2 motif-containing factors (*e.g*. Paip1, Paip2, eRF3, Ataxin-2, Tob2, PAN3 and GW182), which play key roles in regulation of polyadenylation, deadenylation, translation initiation and translation termination (24). LARP4A will compete with these proteins for MLLE binding, and it has been reported that the association of LARP4A with PABP via both the PAM2w and the PBM motif is essential for its described role in net lengthening of the 3′ polyA tail of heterologous mRNA (3).

Altogether, LARP4A plays a central role in many cellular processes. Nonetheless, the mechanistic details of how LARP4A interacts with its partners, including RNA substrates, are poorly understood. To improve our knowledge of LARP4A function and mode of action, we initiated structural and functional studies of its N-terminal domain (NTD) that binds oligoA RNA and comprises the La-module and the PAM2w. These investigations unexpectedly revealed that the La module is not the main site of oligoA interaction, thereby uncovering a novel RNA-binding domain that does not share sequence homology with the traditional canon of domains. They also reveal that LARP4A PAM2w motif mediates both RNA and PABP binding. To understand in detail the molecular mechanisms involved in these processes, we set out to determine by biochemical and biophysical methods whether PABP and oligoA RNA can bind simultaneously to LARP4A. As an outcome, we define two mutually exclusive LARP4A–MLLE and LARP4A–oligoA15 complexes, which likely act in different stages of translational control and may have implications for mRNA stabilization by LARP4A.

## MATERIALS AND METHODS

### Cloning

Human LARP4A deletion mutants were amplified by polymerase chain reaction (PCR) and cloned as hexahistidine-tagged proteins with a TEV (Tobacco etchy virus) protease-cleavage site after the tag. LARP4A (1-287) (called NTD), LARP4A (1-111) (called NTR), LARP4A 24–287, LARP4A 50–287, LARP4A 79–287 and LARP4A 95–287 were inserted into a pRSFDuet-1 vector (Novagen), whilst LARP4A LaM (111–196), RRM1 (196–287) and La module (111–287) into pETDuet-1 (Novagen). The double mutant NTD L15AW22A was amplified by PCR from the full-length HsLARP4A L15AW22A construct (1), gift of Richard Maraia (NIH, USA), and cloned into pRSFDuet-1. The PAM2w single mutants, L15A, W22A and W22F, were generated from the NTD using the Q5 Site-Directed Mutagenesis Kit (NEB). A set of LARP4A mutants was also cloned with a hexahistidine-SUMO tag at the N-terminal, including NTD, LARP4A 1–50, LARP4A 1–79 and NTR. The plasmid pET28SUMO from Christopher Lima (Cornell University, USA) was given to us by Karen Lewis (Texas State University, USA). LARP4A La module C130YM160F mutant was a gift from Mark Bayfield (York University, Canada). PABPC1 MLLE (544–626) construct cloned in pGEX-6P-1, with a GST tag at the N-terminus (1), was a gift from Kalle Gehring (McGill University, Canada). The same fragment was inserted into a pET28SUMO vector to obtain the His-SUMO-MLLE variant.

### Protein expression and purification

All proteins were expressed in *Escherichia coli* Rosetta II cells grown in LB medium. ^15^N- and ^13^C-labelled samples for NMR were grown on minimal media supplemented with 1 g/l of ^15^NH_4_Cl and 2 g/l of uniformly ^13^C-labelled glucose. The cultures were induced with 1 mM of IPTG (isopropyl β-d-1-thiogalactopyranoside) at OD_600_ 0.6 and left growing at 18°C overnight. Cells were harvested and lysed by sonication in a buffer containing 50 mM Tris, pH 8, 300 mM NaCl, 10 mM imidazole, 5% glycerol, protease inhibitor cocktail (Complete tablets, Roche), 2 mM phenylmethylsulfonyl fluoride (PMSF) and lysozyme. All the His-tagged proteins were purified on a 5 ml His-Trap (GE Healthcare) affinity column with gradients varying from 0 to 300 mM of imidazole. The purified samples were incubated with His-tagged TEV protease at 4°C overnight, whilst they were dialysed in a buffer containing 50 mM Tris pH 7.25, 100 mM KCl, 0.2 mM ethylenediaminetetraacetic acid (EDTA) and 1 mM Dithiothreitol (DTT). The proteins with the His-SUMO tag were incubated with His-tagged ULP1 protease instead of TEV during dialysis. The pET28-ULP1 was a gift from Christopher Lima (Cornell University) via Karen Lewis (Texas State University). The mixtures were then loaded onto a Nickel affinity column (Generon) to separate the proteins from the cleaved tags, the protease and non-digested products. The proteins were further purified to eliminate any nucleic acid contamination on 5 ml Hi-Trap Heparin or DEAE columns (GE Healthcare) with gradients of 0 to 1 M KCl. Purified proteins were dialysed in a final buffer containing 20 mM Tris pH 7.25,100 mM KCl, 0.2 mM EDTA and 1 mM DTT.

GST-MLLE (544–626) was purified using Glutathione Superflow Agarose (Thermo Scientific) in PBS buffer (19 mM Na_2_HPO_4_ 0.9 mM KH_2_PO_4_, pH 7.0, 2.5 mM KCl, 140 mM NaCl), and the GST tag was cleaved after overnight incubation with HRV (Human Rhinovirus) 3C Protease. The His-tagged 3C protease was removed in a Nickel affinity column step, and the MLLE was separated from the GST tag by Size Exclusion chromatography in 50 mM Tris pH 7.25, 100 mM KCl and 1 mM DTT.

Protein concentrations were calculated upon the near-ultraviolet (UV) absorption at 280 nm using theoretical extinction coefficients derived from ExPASY (25).

### RNA sample preparation

oligoA15 (5′-AAAAAAAAAAAAAAA-3′) and 5′FAM-oligoA20 (5′-Fluorescein-AAAAAAAAAAAAAAAAAAAA-3′) were synthesized by IBA GmbH (Germany). The lyophilized powder was resuspended in diethyl pyrocarbonate (DEPC)-treated water to a final concentration of 2 mM. RNA samples were diluted to lower concentrations in the suitable buffers for the next experiments.

### NMR spectroscopy

The ^15^N- and ^15^N,^13^C-labelled samples of LARP4A La module (111–287), LaM (111–196) and RRM1 (196–287) were concentrated to 400–600 μM in a buffer containing 20 mM Tris, pH 7.25, 100 mM KCl, 0.2 mM EDTA and 1 mM DTT. All the NMR experiments were performed at 25°C on Bruker Avance or NEO NMR spectrometers operating at 700, 800 and 950 MHz, equipped with triple resonance cryoprobes.

The ^1^H, ^13^C and ^15^N resonance assignments of hLARP4A LaM, the RRM1 and the La module were obtained manually and have been reported elsewhere (26). For structure calculation, ^1^H/^15^N- and ^1^H/^13^C-edited NOESY-HSQC experiments were performed. T1, T2 and {^1^H}-^15^N heteronuclear NOE relaxation experiments were recorded using the pulse sequences adapted from standard schemes and analysed with CcpNMR analysis (27). The samples were prepared at a final concentration of 250 μM in the NMR buffer described above.

For the analysis of ^1^D_NH_ residual dipolar couplings, LARP4A La module (111–287) was aligned in a medium containing 15 mg/ml of Pf1 filamentous phages (Asla Biotech) in 20 mM Tris, pH 7.25, 100 mM KCl, 0.2 mM EDTA and 1 mM DTT with 10% of D_2_O. This liquid crystalline medium produced a stable quadrupolar splitting of the D_2_O signal of ∼15 Hz at 25°C. The final concentration of the protein was 280 μM. The analysis was performed by comparing ^1^H-^15^N HSQC spectra of the protein in the presence or in the absence of Pf1 phages.

For RNA titration experiments, oligoA15 was titrated into a sample of La module (111–287) at a concentration of 200 μM up to a 1:1.4 molar ratio of protein:RNA (in 20 mM Tris, pH 7.25, 100 mM KCl, 0.2 mM EDTA and 1 mM DTT). For the NTD, 800 μl of protein at a concentration of 50 μM was slowly added to 200 μl of oligoA15 at 270 μM (protein:RNA molar ratio of 1:1.2) in 20 mM Tris, pH 7.25, 100 mM KCl, 0.2 mM EDTA and 1 mM DTT. The mixture was concentrated in a 3 kDa cutoff centrifugal filter (Amicon Ultra, Millipore) at 4°C and 10 000 *g*, reducing the volume to 300 μl. The final concentration of the protein–RNA complex sample was ∼150 μM.

Chemical shift perturbation of NH resonances between different protein fragments and in absence/presence of RNA was obtained by comparing ^1^H-^15^N HSQC spectra. The average chemical shift differences Δδ_avg_ were calculated as [0.5 [Δδ(^1^H_N_)^2^ + 0.2 Δδ(^15^N)]^2^]^1/2^ with Origin Pro8 (OriginLab).

The CLEANEX-PM experiments (28) on LARP4A La module and NTD were collected at 950 MHz with a 3-s acquisition delay and mixing times of 1, 10, 20, 39, 59, 79 and 100 ms using standard Bruker pulse programs. Backbone amide protons that exchanged with the solvent were fitted using established methods (28) with NMRPipe (29), and the exchange rates were calculated with Origin Pro8 (OriginLab).

All the spectra were processed using Topspin 3.5pl7 software (Bruker) and NMRPipe/NMRDraw (29), the assignment and the analysis of the resonances were performed with CcpNMR analysis (27) and/or CARA/NEASY (30).

### Structure calculation

Structures were calculated using UNIO software, based on ATNOS/CANDID algorithms (31,32) for cross-peak detection and structure determination. For the individual LARP4A LaM and RRM1 domains, an almost complete backbone and side-chain resonance assignments and three 3D heteronuclear-resolved [^1^H,^1^H]- NOESY experiments were provided as input. Automatic NOE assignments and structure calculations were performed by UNIO coupled to XPLOR-NIH (33).

Structure calculation of LARP4A La module was performed using the individual motifs as starting point, inputting assignments into UNIO to derive automatic NOE assignments from NOESY spectra recorded in the La module. Subsequently, NOEs were manually checked and applied as distance restraints together with dihedral angle restraints and hydrogen bond restraints in a simulated annealing protocol using CNS (34). Dihedral restraints were derived from TALOS+ (35), and hydrogen bond distance restraints were applied based on secondary structure identified by NOEs and dihedral angles. Ribbon representations and the electrostatic surface potential were prepared with PYMOL (DeLano Scientific, San Carlos, CA, USA) and MOLMOL (36), respectively.

Calculation was performed using 1051 intraresidue restraints, 542 sequential (*i, i* + *j, j* = 1), 314 short range (*i, i* + *j* (*j* ≤ 4) and 422 long range *i, i* + *j* (*j* > 4). 6 H-bonds, 154 ϕ and 154 φ dihedral angles. The final family, comprising 20 structures of lowest total energy from a total of 300 calculated structures, was inspected using Procheck-NMR (37), PSVS (38) and wwPDB Validation tool.

### Electrophoretic mobility shift assay (EMSA)

RNA oligoA15 (5′-AAAAAAAAAAAAAAA-3′) was labelled at the 5′ end with γ-^32^P ATP using T4 polynucleotide kinase. The unincorporated nucleotides were removed on G-25 spin columns (GE Healthcare). The binding reactions were performed in 20 mM Tris, pH 7.25, 200 mM KCl, 5% glycerol, 1 mM DTT, 0.1 mg/ml bovine serum albumin (BSA) and 0.01 mg/ml of unlabelled *E. coli* MRE 600 tRNAmix in a total volume of 22 μl. Parallel electrophoretic mobility shift assays (EMSA) experiments were conducted in the absence of competitor tRNA to assess RNA-binding specificity of LARP4A proteins. The final concentration of ^32^P-labelled oligoA15 in each reaction was 2 nM. The RNA was incubated for 10 min on ice with LARP4A mutants, at concentrations ranging from 200 to 1.6 μM or from 20 to 0.15 μM by serial (1:1) dilutions. After the addition of 2 μl of 30% Ficoll, 6 μl of each reaction was loaded on a 9% native polyacrylamide gel prerun at 100 mV for 1 h at 4°C in 0.5 × TBE (Tris-borate-EDTA buffer). Each EMSA experiment was run at 4°C for 1 h at 125 mV in 0.5 × TBE. Gels were dried onto 3MM chromatography paper and then exposed to a phosphoimaging plate overnight at room temperature. The intensity associated with each band was measured with the phosphoimager Typhoon Trio and quantified with Image Quant TL software. The fraction of bound RNA was plotted versus the protein concentrations. To determine the dissociation constants (*K*_D_), data were fitted with Origin Pro8 (OriginLab) to a sigmoidal binding curve using the modified Hill equation described in (39) to compensate for deviations from ideal conditions.

For a subset of experiments, EMSA were performed using an RNA oligoA20 labelled at the 5′ end with 5-FAM. The protein–oligoA reactions were performed as described above using 5′FAM-oligoA20 at a final concentration of 10 nM and protein concentrations ranging from 200 to 1.6 μM by serial (1:1) dilutions. The native gels were visualized with the ChemiDoc MP imager (Biorad) using the Epi Blue-Light module for excitation in combination with the 530/28 nm filter to measure the emission. The fraction of bound RNA was plotted versus the protein concentrations. To determine the dissociation constants (*K*_D_), data were fitted with Origin Pro8 (OriginLab) using the modified Hill equation described in (39). The average values for *K*_D_ and standard deviations reported were calculated from at least three biological and three technical replicates. Error bars have been indicated in all cases.

### Competition EMSA experiments

Quantitative competition-binding experiments were carried out titrating PABP MLLE protein ranging from 12.5 to 200 μM into a pre-formed LARP4A NTD–oligoA complex prepared by incubating 7.5 μM LARP4A NTD and 2 nM of ^32^P-labelled oligoA15. The competition constant *K*_C_ was calculated by plotting the fraction of bound RNA versus the concentration of competitor and fitting the curve as described in (39).

### Pull-down assays

His-SUMO-MLLE (200 μg) was incubated with 250 μg of different LARP4A proteins on ice for 5 min in a total volume of 300 μl. The protein mixture was then added to 200 μl of Super Ni-NTA Agarose Resin (Generon) and incubated on ice for another 5 min. The beads were washed seven times with 500 μl of a buffer containing 50 mM Tris, pH 7.25, 100 mM KCl, 0.2 mM EDTA, 1 mM DTT and 10 mM imidazole, and then eluted with 500 μl of the same buffer containing 500 mM imidazole. All the steps to separate the supernatant from the resin were performed by centrifugation at 7500 *g* for 1 min at 4°C.

### Circular dichroism (CD) spectroscopy

UV and CD spectra of proteins were acquired on the Applied Photophysics Chirascan & Chirascan Plus spectrometers (Leatherhead, UK) using Suprasil rectangular cells of 10 and 0.5 mm path lengths (Starna Scientific Ltd) in the region of 400 to 190 nm under constant nitrogen flush. The final concentration of the samples was 0.2 mg/ml in a buffer containing 20 mM Tris, pH 7.25, 100 mM KCl, 1 mM DTT. The experiments were run at 25°C with 2 nm spectral bandwidth, 1 nm data step-size and 1.5 s time-per-data-point. UV spectra were acquired in the region from 400 to 230 nm to determine the exact concentration of the proteins from the absorbance value at 280 nm. CD spectra were performed from 260 to 190 nm, and the data in mdegrees were converted to mean residue ellipticity [θ] (deg·cm^2^·dmol^−1^) (40). All far-UV CD spectra were processed using Savitsky-Golay smoothing with a convolution width of 4 points. The secondary structure content was estimated with BeStSel server (41).

### Microscale thermophoresis (MST)

The MST experiments were performed at 50% of LED power and 20% of MST power on a Monolith NT.115 instrument (Nanotemper Technologies) at 25°C with standard capillaries. The binding reactions were prepared in a buffer containing 20 mM Tris, pH 7.25, 100 mM KCl, 0.2 mM EDTA, 1 mM DTT and 0.05% Tween-20 in a total volume of 20 μl. The final concentration of 5′FAM-oligoA20 was 25 nM and the protein concentrations ranged from 200 to 0.03 μM by 1:1 serial dilutions. The dissociation constant was determined using the region of thermophoresis alone and the data fitting to a non-linear binding curve (modified Hill equation) was performed with Origin Pro8 (OriginLab).

### Protein sequence alignment

Sequence alignments were performed using Clustal Omega (42) Uniprot portal (http://www.uniprot.org/align/). The alignments were edited and analysed with Jalview software (43).

### Disorder/secondary structure prediction and functional motif searches

The web servers JPred (http://www.compbio.dundee.ac.uk/jpred/) (44), PredictProtein (https://www.predictprotein.org/) (45), IUPred (https://iupred2a.elte.hu/) (46), FoldIndex (https://omictools.com/foldindex-tool) (47) and DISOPRED (48) were used to predict the ordered and disordered regions of LARP4A. A search for functional motifs was performed with the eukaryotic linear motif (ELM) resource (49). EMBOSS Needle tool (50) was used for the pairwise alignments of LARP4A sequence with disordered protein regions described to bind RNA (51).

### Native mass spectrometry

About 10 μM of purified LARP4A NTD was mixed with RNA oligoA15 at a 1:1 molar ratio and incubated on ice for 5 min. LARP4A and LARP4A-oligoA were then buffer exchanged into MS buffer (200 mM ammonium acetate, at pH 7.25) using micro Bio-Spin Chromatography columns (Micro Bio-Spin 6 Columns, Bio-Rad). A total of two washes were performed. Prior to MS analysis, 3 μl aliquots of the sample were directly infused via nano electrospray using gold-coated borosilicate capillaries. A Synapt G2Si High Definition MS system (Waters) was used to record all spectra. Instrument settings were 1.3–1.7 kV capillary voltage, 30–50 V collision voltage, 20–50 V cone voltage, extraction voltage of 8 V, a transfer voltage of 2 V and bias voltage of 30 to 35 V. The source pressure was 5–7 mbar, and the source temperature was 20–25°C. The buffer gas helium was 2.1 Torr. Drift cell gas was N_2_ (pressure of 1.6 Torr), and the collision gas was argon (5–8 ml min^–1^).

## RESULTS

### LARP4A La module folds into two domains lacking a rigid relative orientation

The 3D structure of the La module of human LARP4A was determined using standard heteronuclear multidimensional NMR techniques. These analyses showed that, analogously to other LARPs, LARP4A La module comprises two independently folded globular domains, the LaM and the RRM1, connected by a linker (Figure 1).

The relative orientation of the LaM and RRM1 is undefined. No unambiguous contact between the two domains and/or the linker could be detected and no fixed orientation could be found in our investigations. This is consistent with backbone relaxation analysis where R2/R1 ratios of residues in the La module are lower than would be expected for a single domain of the same size (Supplementary Figure S1). Further evidence originated from ^1^D_NH_ residual dipolar coupling measurements in liquid crystalline media. Of the several systems tried, the only suitable medium that did not affect the La module stability was Pf1 phages, in which however all the NH signals from the RRM1 domain disappeared or weakened significantly, leaving the rest of the resonances seemingly unaffected (Supplementary Figure S2). This suggests that the positively charged RRM1 (Supplementary Figure S3) preferentially interacts with the Phage orientating medium, resulting in a significantly greater degree of alignment for this domain compared to the LaM and the C-terminal tail (beyond residue 274) (52). These data support the view that the two domains do not adopt a rigid relative orientation in solution.

An ensemble of 20 final lowest energy structures was obtained from the structural calculation (Figure 1B–D). Structure statistics, restraints violation and deviation from the ideal geometry are given in Table 1, and a representative structure is reported in Figure 1E. Each domain was overlaid separately: the overall values of pairwise root mean square deviation (rmsd) between the family and the mean coordinate position are 0.45 and 0.37 Å for all backbone atoms for the LaM (residues 120–195) and the RRM1 (residues 200–274) respectively, and 0.81 and 0.89 Å for all heavy atoms respectively (Table 1).

**Table 1.** Summary of structure statistics for LARP4A La module

*NMR restraints*	
*Distance restraints*	
Total NOE	2335
Intraresidue	1051
Inter-residue	
Sequential (*i, i* + *j, j* = 1)	542
Short range (*i, i* + *j, j* ≤ 4)	314
Long range (*i, i* + *j, j* > 4).	422
Hydrogen bonds	6
*Dihedral angle restraints*	
ϕ	154
ψ	154
*Structure statistics*	
Average pairwise rmsd (Å)*	
Backbone (LaM; RRM1)	0.45; 0.37
Heavy (LaM; RRM1)	0.81; 0.89
*Violations (mean ± s.d.)*	
Distance restraints (Å)	0.019 ± 0.001
Dihedral angles restraints (°)	0.354 ± 0.026
*Deviations from idealized geometry*	
Bond lengths (Å)	0.002 ± 0.000
Bond angles (°)	0.360 ± 0.004
Impropers (°)	0.246 ± 0.004
*Ramachandran statistics of 20 structures (Procheck NMR)*	
Percentage residues in	
Most favoured regions	81.8%
Additional allowed regions	17.1%
Generously allowed regions	1.1%
Disallowed regions	0.0%

*Residues selected on the basis of ^15^N backbone dynamics. LaM: 120–195; RRM1: 200–274.

The LaM of LARP4A adopts the same elaborated winged-helix domain fold that was discovered in La and described in detail elsewhere (2,12,53), consistent with the 49% amino acid sequence similarity with La. Superposition of the LaM of LARP4A and La revealed small but notable differences in the length of helices α1 and α2, both being shorter in LARP4A (Supplementary Figure S4), reflected in the ∼1.9 Å pairwise rmsd in backbone atom positions between chains. This however excludes the main element of dissimilarity, namely the C-terminal structured loop extending from strand β3 known as ‘wing2’ in winged helix domains, that is totally absent in LARP4A. Interestingly, wing2 variations have been hitherto noted as a divergent feature of the LaM fold of LARPs (2,17). Moreover, the sequence VQVDEKGEKVRP at the C-terminal boundary of the LaM is almost entirely conserved in the PAM2-containing LARP4 family members (11), and this is likely to bear structural repercussions on the overall La-module architecture and conformation (see below).

The RRM1 of LARP4A adopts a canonical RRM-like fold consisting of four antiparallel β-strands that pack together to create a centrally located platform, flanked on one side by two α-helices (54). RRM1 domains within the LARP4 family retain a high degree of sequence conservation, in contrast to LARP6 proteins (17), and all lack the conserved RNP1 and RNP2 aromatic residues residing on the β3 and β1 strands respectively of classical RRMs (11). The central β-sheet platform of LARP4A RRM1 overlays well with that of human La RRM1, although comparison of the two structures shows significant variability in the conformation and lengths of the α-helices and the surface exposed loops, particularly the loops β1-α1 and β2-β3. LARP4A also lacks the C-terminal helix present in La, albeit it is appended by a flexible C-terminal tail spanning residues 274–287 (Supplementary Figure S4).

The interdomain linker of the La module is another divergent trait of LARPs that was previously unveiled (17). Consistent with this, our structure precisely maps the LaM-RRM1 linker of human LARP4A to a 3-residue stretch interconnecting the two domains (^197^HKR^199^), which is specific and invariant for LARP4A and invertebrate LARP4 proteins (11) but not present in any other LARP, nor in LARP4B members where it diverges into Q(N/S)R. In our structure, the interdomain linker adopts an extended conformation: the resonance assignment is missing for residues 196–198 and no NOE contacts could be detected with residues of either the LaM or the RRM1.

Although our structure indicates that in the apo form the LaM and RRM1 are not in a rigid tandem domain conformation, the short interdomain linker coupled with the absence of wing2 give rise to a somewhat elongated configuration for LARP4A (Figure 1). This is also in agreement with SAXS data (Conte *et al.*, in preparation). It was speculated that this would be consistent with a binding requirement of a longer single-stranded RNA (*e.g*. 15nt oligoA) compared with the 3–4 nt target of hLa (2), given that La is able to adopt a distinct V-shaped topological arrangement to clamp the short RNA ligand in the protein-binding crevice (2,13).

### LARP4A NTD is the minimal oligoA RNA-binding region

To investigate whether the elongated LARP4A La module structure was a determinant for RNA-binding specificity and affinity, EMSA were performed.

**Figure 2.**
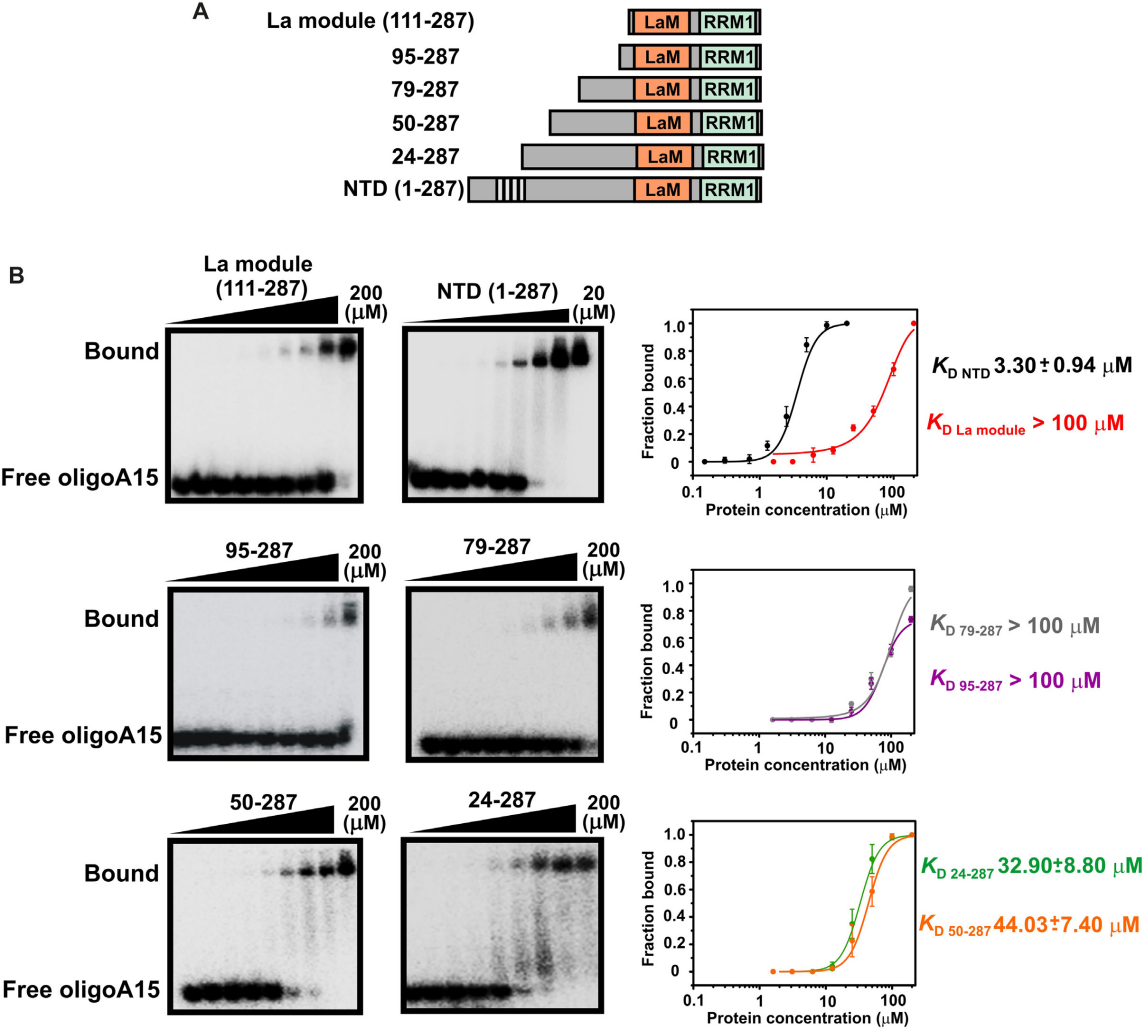
The N-terminal domain of LARP4A binds to oligoA15 RNA. (**A**) LARP4A deletion mutants used in the experiments. (**B**) EMSA binding assays of LARP4A–NTD and its N-terminal truncation mutants (La module, 95–287, 79–287, 50–287 and 24–287) with ^32^P-oligoA15. Representative autoradiograms for La module (left, top), 95–287 (left, middle), 50–287 (left, bottom), NTD (centre, top), 79–287 (centre, middle) and 24–287 (centre, bottom). For the NTD, protein concentrations of 0, 0.15, 0.3, 0.7, 1.3, 2.5, 5, 10 and 20 μM were used; for the deletion mutants, the concentrations were 0, 1.6, 3.1, 6.3, 12.5, 25, 50, 100 and 200 μM. Bound and free RNA populations are labelled. Right: Binding curves showing fractions of protein-bound RNA plotted as a function of protein concentration and the fitting of the data. Each curve is colour labelled. Average values for *K*_D_ (dissociation constant) and standard deviations were calculated from at least three biological replicates, and error bars have been reported. It is noteworthy that quantification of binding interactions by EMSA is subject to experimental deviations from ideal conditions, including binding reactions not reaching equilibrium, incomplete binding due to inactive populations, aggregation and/or proteins/RNA sticking to apparatus (39).

Human LARP4A binds to the oligoA tails of mRNA. *In vitro*, a single-stranded stretch of minimum 15 adenines is required for maximum affinity binding (1). Although most of previous investigations were performed using the entire NTD of LARP4A, comprising the structured La module core and a 110-residue long N-terminal region (Figures 1 and 2A), a major role of the La module in RNA binding was anticipated. Unexpectedly, EMSA experiments showed a weak binding affinity of LARP4A La module for this RNA target, with a dissociation constant (*K*_D_) estimated between 100 and 200 μM (Figure 2B). This prompted us to perform comparative EMSA analyses with the entire LARP4A NTD (spanning residues 1–287). In agreement with previous studies (1), LARP4A NTD displayed low micromolar affinity for oligoA15 — more than 100-fold tighter than the La module alone (Figure 2B). This experiment concluded that high affinity interaction with oligoA15 RNA requires the N-terminus of LARP4A.

To map the extent of the N-terminal region needed for high affinity association with oligoA, we made systematic truncations of the LARP4A NTD, namely 95–287, 79–287, 50–287 and 24–287 (Figure 2B), adding N-terminal tails of variable length to the conserved La module. Although gradual inclusion of N-terminal residues provided progressively tighter RNA binding, none of the truncation mutants recapitulated the behaviour of the full NTD (Figure 2).

### LARP4A N-terminal region contains the principal determinants for oligoA recognition

**Figure 3.**
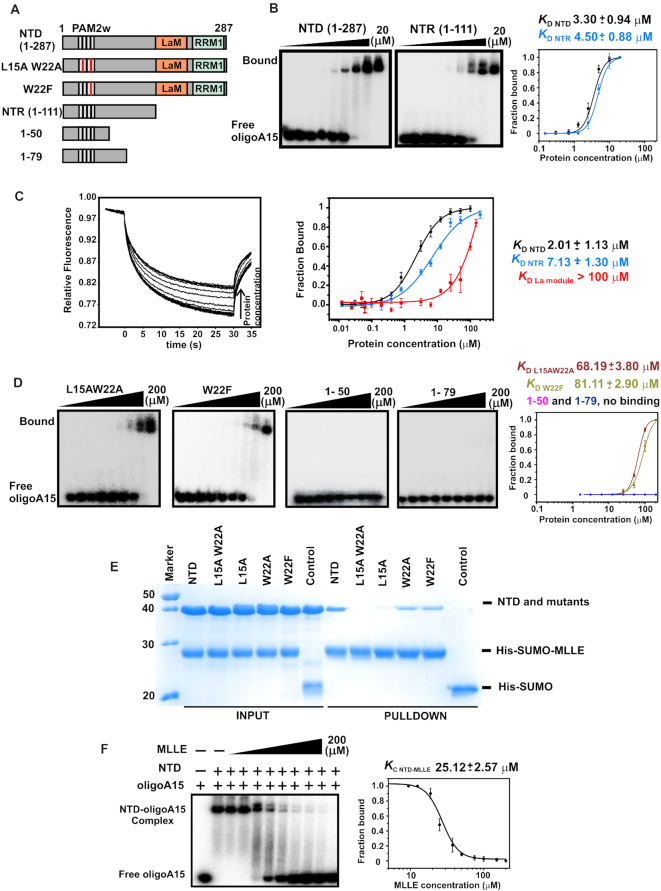
LARP4A N-terminal region dominates oligoA15 binding, with the PAM2w motif playing a key role. (**A**) Schematic of LARP4A mutants used in the experiments. (**B**) EMSA binding assays of LARP4A–NTD and NTR with ^32^P-oligoA15. Representative autoradiograms are shown for NTD (left) and NTR (centre), and their binding curves (right). Curve fitting generated the reported *K*_D_. The protein concentrations used were 0, 0.15, 0.3, 0.7, 1.3, 2.5, 5, 10 and 20 μM. Bound and free RNA populations are labelled. (**C**) MST data and analysed binding curves for the interaction of LARP4A NTD, NTR and La module with 5′FAM-oligoA20. Left: Normalized thermophoretic time-traces from one representative 16-sample experiment for LARP4A NTD. Right: Binding plots showing the fractions of protein-bound RNA as a function of protein concentrations for the three mutants tested. The lines represent the fitting of the data using a modified Hill equation, giving a 1:1 protein:RNA molar ratio. Error bars have been reported. (**D**) EMSA binding assays of LARP4A NTD deletion and PAM2w mutants with ^32^P-oligoA15. Representative autoradiograms of mutants L15AW22A, W22F, 1–50, 1–79 and their binding data plots are shown from left to right. Curve fitting generated the reported *K*_D_; for these mutants the protein concentrations were 0, 1.6, 3.1, 6.3, 12.5, 25, 50, 100 and 200 μM. Bound and free RNA populations are labelled. The average values of the *K*_D_ and the standard deviations reported in (B–D) were calculated from at least three replicates for each experiment. Each curve is colour labelled. (**E**) SDS-PAGE analysis of Nickel affinity pull-down assays to analyse the interaction between LARP4A PAM2w mutants to MLLE. Untagged LARP4A NTD and mutants L15AW22A, L15A, W22A and W22F were purified and mixed with purified His-SUMO-MLLE, used as bait. Pull-down assays were carried out using NiNTA affinity beads in a buffer containing 100 mM KCl. The Coomassie-stained 12% SDS-PAGE gel shows the input fractions applied to the resin and the pulled-down proteins eluted with 500 mM imidazole. Control experiments with His-SUMO alone exclude unspecific binding to the tag, and a representative control experiment performed with NTD is shown in the lane labelled ‘control’. (**F**) Competition binding experiments between LARP4A NTD, PABPC1 MLLE and oligoA15. Left: Competition mobility shift experiments titrating MLLE into a pre-formed complex of NTD-^32^P-oligoA15. MLLE concentrations used were 0, 12.5, 18.8, 25, 37.5, 50, 75, 100, 150 and 200 μM. Right: Plot and fitting of the data extracted from left. A fit of the NTD–oligoA fraction bound versus competitor MLLE concentration provides a measure of the affinity of MLLE for NTD. The equilibrium dissociation constant of the competitor (*K*_C_) and the standard deviation were calculated from at least three replicates of the experiment. The *K*_C_ is equivalent within error to the *K*_D_ previously determined by direct titration (1).

Given the drastic reduction in RNA-binding ability of the truncation mutants, we sought further clarification on the relative importance of the La module and the N-terminal region (NTR) to RNA binding. Defying expectations, EMSA experiments comparing the behaviour of the isolated halves (NTR, encompassing residues 1–111 and La module 111–287, Figure 1) versus the entire NTD revealed that the NTR is the main hotspot for this association, retaining the majority of the binding affinity for oligoA (Figure 3B and Supplementary Figure S5). Maximum affinity is nonetheless achieved when the NTR and the La module are tethered in the context of the NTD, suggesting that the LARP4A NTD–oligoA complex is stabilized somewhat by additional contacts with La module. EMSA results for LARP4A NTD, NTR and La module were confirmed by microscale thermophoresis (MST) (Figure 3C): the binding constants derived from these analyses are in agreement with one another, indicating that both techniques are following the same process. MST methodology also affords a quantitative analysis of the interactions, given that equilibrium dissociation constants in the micromolar range cannot be accurately determined by standard EMSA (39). A 1:1 protein:RNA stoichiometry was obtained by native mass spectrometry (Supplementary Figure S6).

Taken together, these data argue for the formation of a LARP4A NTD–oligoA15 complex with the N-terminal regions and the La module both establishing contacts with the RNA, albeit having a significantly different contribution to the association.

### The PAM2w motif plays a key role in oligoA RNA binding

Although the EMSA experiments with the N-terminal truncation mutants suggested some involvement of the stretch spanning amino acids 24–79 to oligoA RNA association (Figure 2), regions within the first 24 residues clearly contributed to a greater effect to this molecular recognition. Intriguingly, the PAM2w motif is located herein (1), encompassing residues 13–26. It is known that the double mutation L15AW22A abrogates the interaction between LARP4A PAM2w motif and the MLLE domain of PABPC1 by disrupting key hydrophobic interactions (1); we asked whether this also affected the association with oligoA RNA. Unexpectedly, L15AW22A mutant displayed a severely impaired RNA-binding ability (Figure 3D). To probe these interactions in greater depth, single mutants L15A and W22A were also assayed, alongside a W22F mutant, designed to replace the variant tryptophan residue of LARP4A PAM2w motif with phenylalanine, which is the otherwise conserved amino acid in this position in consensus PAM2 motifs (24). Interestingly, all of the PAM2w mutants tested displayed low binding affinity for oligoA15, underscoring the importance of this stretch to RNA binding (Figure 3D and Supplementary Figure S7). To our knowledge, this is the first example of a functional PAM2 motif that was also revealed to be essential in mediating protein–RNA interactions (see below).

In parallel, the PAM2w mutants were also probed for their ability to bind PABP. A PABPC1 MLLE domain fused with a His-tagged SUMO tag (His-SUMO-MLLE) was used as bait in pull-down assays with LARP4A NTD and its PAM2w mutants performed on a nickel affinity resin (Figure 3E). Single substitution of W22 with A or F did not significantly impair protein–protein interaction, whilst, consistently with previous findings (1), MLLE binding of L15A and L15AW22A mutants was reduced and undetectable, respectively. No interaction was observed with His-SUMO alone (Figure 3E).

Altogether, these studies implicate the PAM2w motif of human LARP4A in RNA interaction and provide a mechanistic explanation of the presence of the tryptophan in the LARP4 proteins instead of the otherwise conserved phenylalanine (see below). Given the critical importance of the PAM2w motif to oligoA RNA binding, and to narrow down further the principal interacting region within the NTR, we next interrogated the RNA-binding behaviour of shorter PAM2w-containing fragments, encompassing residues 1–50 and 1–79. Interestingly, these fragments failed to bind (Figure 3D). These results indicate that a contiguous region at the N-terminus of LARP4A is required for oligoA RNA binding. Consistent with this, the high degree of aminoacid sequence conservation in the NTR region of vertebrates underscores its functional importance for LARP4A proteins (Supplementary Figure S8).

### The oligoA binding by LARP4A is mutually exclusive to PAPB MLLE interaction

Thus, LARP4A NTD engages oligoA RNAs at the surface containing the PAM2w motif, which also mediates the interaction between LARP4A and the MLLE domain of PAPB, raising the question as to whether the interactions of LARP4A with PABP MLLE and RNA are concomitant or mutually exclusive. To address this question we used EMSA competition assays, by titrating increasing concentrations of MLLE to a preformed LARP4A NTD–oligoA complex (39). No formation of a ternary complex was observed. The lack of simultaneous binding was confirmed by the ability of the MLLE domain to trigger dissociation of the RNA from LARP4A (Figure 3F). The equilibrium dissociation constant of the competitor MLLE domain was determined (39), and this conforms exceptionally well with the direct binding constant previously reported (1). These experiments show that the interaction of LARP4A NTD is mutually exclusive with either MLLE domain or oligoA.

### An unconventional RNA-binding surface comprising intrinsically disordered protein regions

Our studies have identified a new RNA-binding mode of LARP4A NTD, dominated by the NTR. What is the molecular basis for such recognition? A bioinformatics search using several protein domain databases did not identify known functional or structural motifs within the N-terminal 110 residues of LARP4A, beyond the PAM2w. No obvious RNA-binding motif was found. Furthermore, a degree of disorder was predicted for this region by several computational meta-servers, albeit ordered stretches, possibly containing secondary structure elements, were forecast in the N-terminal 20 residues and in the region within aa 65–75 (Supplementary Figure S9).

**Figure 4.**
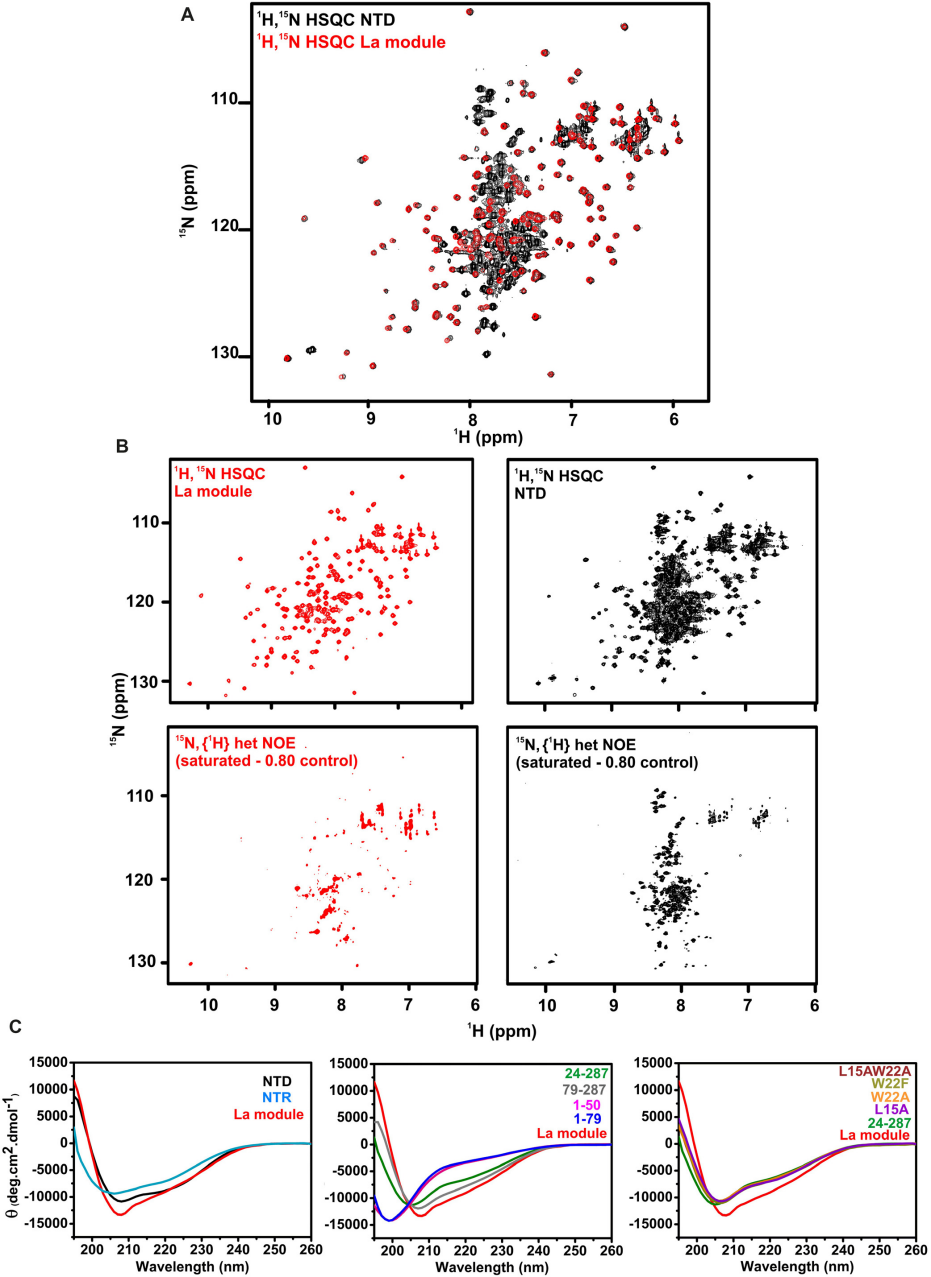
LARP4A NTD contains structured and disordered regions. (**A**) Superimposition of the ^1^H-^15^N HSQC spectra of the La module (red) and the NTD (black). (**B**) Comparison of ^1^H-^15^N HSQC and a subspectrum of {^1^H}-^15^N heteronuclear NOE experiment (saturated - 0.80 control) for La module (top and bottom left panels) and NTD (top and bottom right panels). The signals left in the subtraction spectra on the bottom undergo motions on the pico- to nanosecond timescale. The majority of the NTR resonances are experiencing fast motion. (**C**) Far-UV CD analysis. Far-UV CD spectra of: left, LARP4A NTD, NTR and La module; centre, 24–287, 79–287, 1–50 and 1–79 mutants compared to the La module; right, L15AW22A, W22F, W22A and L15A mutants compared to 24–287 and La module. Each CD trace is colour labelled. The CD profiles indicate that the N-terminal region contains disordered regions and secondary structure elements (see text).

To understand the structural bases of this novel RNA recognition, we embarked on a biophysical characterization of LARP4A NTD. NMR analysis of LARP4A La module versus the NTD allowed us to identify the presence of the globular LaM and RRM1 domains, flanked by a long partially structured N terminus (residues 1–111). This was manifest in the comparative dispersion pattern of amide group chemical shifts and in the dynamic backbone analysis as revealed by {^1^H}-^15^N heteronuclear NOE values (Figure 4A and B). The ^1^H and ^15^N resonances of the La module were easily transferred to the ^1^H-^15^N HSQC of the entire NTD, indicating that the 3D structure of the La module is retained in the context of the longer fragment, although small chemical shift perturbations were noted (see below). The high susceptibility of LARP4A NTD to aggregate forced us to work with a maximum protein concentration of ∼150 μM which, allied to severe spectral overlap, precluded the sequence-specific assignment of N-terminal residues preceding the LaM. Nonetheless, the majority of the amide proton signals arising from the N-terminal fragment could be collectively assigned to a cohort of ∼90 resonances mainly clustering between 7.5 and 8.5 ppm and exhibiting reduced {^1^H}-^15^N NOE values, indicative of disorder (Figure 4A and B).

LARP4A NTD is therefore modular in construction and does not adopt a well-defined single tertiary structure. We were alerted to the possibility of weak transient interactions between the N-terminal arm and the La module by the observation that ∼66% of amide signals in the LARP4A La module experienced chemical shift perturbations in the context of the NTD, albeit of small magnitude (Figure 4A and Supplementary Figure S10). The perturbed residues mapped onto the structure of the La module spread across the entire domain (Supplementary Figure S10), indicating a general effect rather than delineating specific surfaces potentially involved in contacting the N-terminal region.

**Figure 5.**
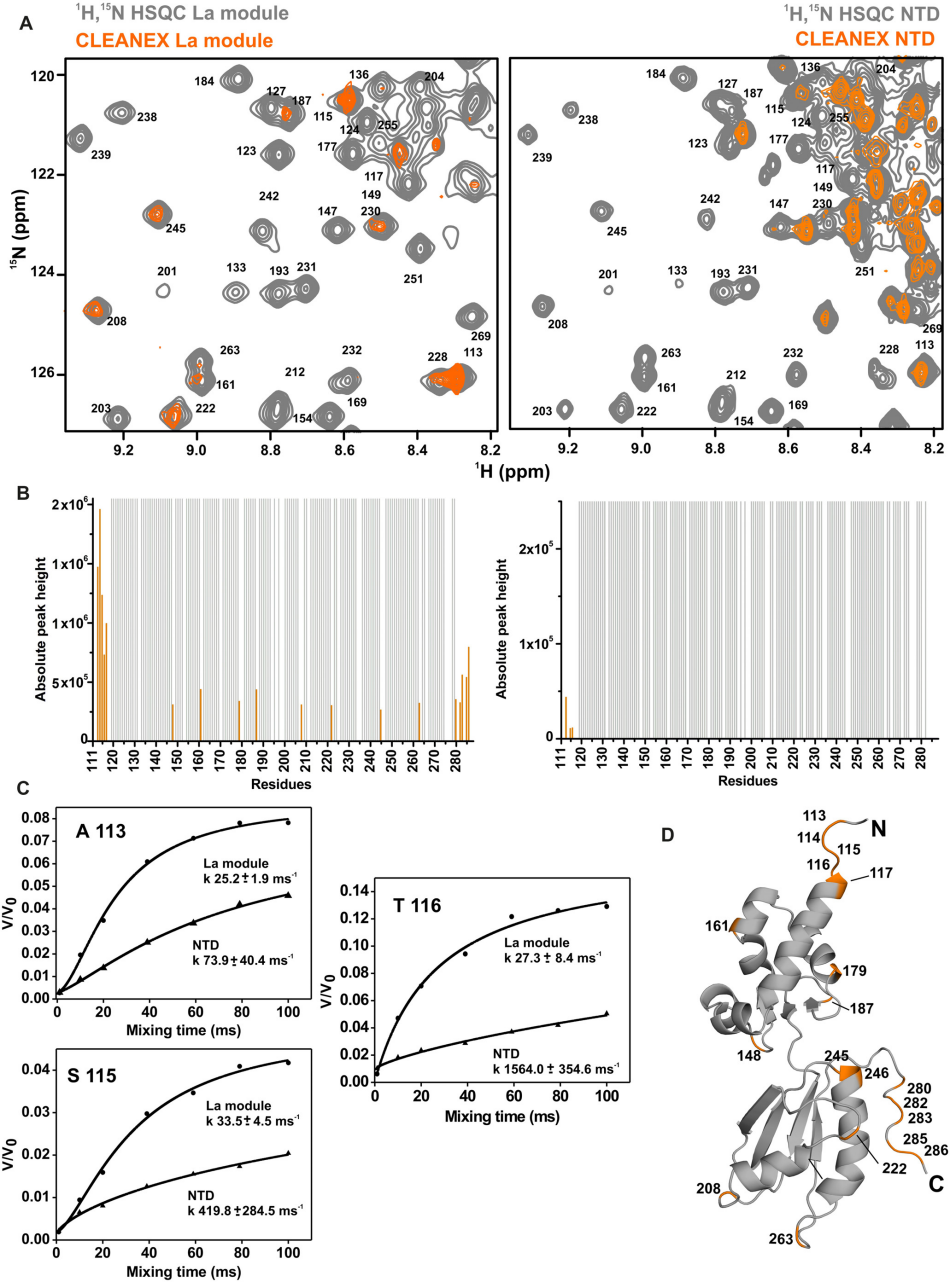
Solvent-exposure of LARP4A La module backbone protons is quenched in the context of the NTD. Analysis of CLEANEX-PM experiment performed on LARP4A La module and the NTD. (**A**) Zoomed-in area of the CLEANEX spectrum recorded with 100 ms mixing time (in orange) superposed over the control ^1^H-^15^N HSQC (in grey) for the La module (left) and NTD (right). The full spectra are shown in Supplementary Figure S10. (**B**) Solvent-exchanging La module residues are indicated with orange bars in the experiment run on the La module in isolation (left), and they are affected in the context of the NTD (right). Absolute peak heights of the exchanging amide protons are reported (in orange), whereas the protected residues appear in grey with arbitrary values. A few residues of the La module (246, 283, 284 and 285) could not be analysed in the NTD experiment due to spectral overlapping and were omitted from the diagram (right panel). (**C**) Fitted solvent-exchange rates in ms^−1^ from CLEANEX analysis for selected amide protons of the La module, comparing data obtained from La module alone or tethered in the intact NTD. (**D**) Solvent-exchanging amides in the La module from panel (C) were coloured in orange and labelled onto a representative structure of the La module.

To understand better the nature of the conformational equilibrium sampled by LARP4A NTD, we appraised the ability of LARP4A NTD backbone amide protons to exchange with the solvent using experiments based on Phase-Modulated CLEAN chemical EXchange (CLEANEX-PM) transfer (28). Measurements performed on the La module alone versus the entire NTD were able to report on changes of residues solvent exposure in the longer versus the shorter fragment. In the La module in isolation, the residues at the N- and C-termini and others in surface exposed loops or at the extremities of α-helices exhibited proton exchange contributions (Figure 5). This is in agreement with the structural and backbone relaxation data showing internal motions for some of these regions. Interestingly, the majority (if not all) of these solvent accessible residues on both LaM and RRM1 experienced a significant reduced water-exchange rate in the context of the entire NTD (Figure 5 and Supplementary Figure S11), indicating that they become protected in the longer protein. This could be rationalized by either displacement of water molecules from the La module by the N-terminal tail or by the N-terminal stretch forming water-mediated interactions that significantly reduce exchange rates (*e.g*. H-bond formation). Considerably more solvent exchange was observed for the majority of resonances assigned to the N-terminal region compared to the La module, demonstrating a greatest degree of solvent accessibility in agreement with the transient equilibrium states postulated by chemical shift and heteronuclear NOE collective data. Nonetheless, a subset of these resonances experienced a comparatively low solvent exchange (Supplementary Figure S11), consistent with the hypothesis of local secondary structure elements and transient contacts within the NTD. Taken together, the chemical shift, backbone relaxation measurements and CLEANEX data indicate that LARP4A–NTD could be best described as an ensemble of rapidly interconverting structures containing a combination of structured domains and flexible regions, and undergoing conformational equilibrium with populated ‘closed’ conformations in which the N-terminal arm folds back onto the La module.

Further details about the structural and RNA-binding properties of the N-terminal arm were derived from far-UV CD analyses: the spectrum of LARP4A NTD differs from that of the La module, displaying a different overall shape and deeper minima around 220–225 nm, features which were retained in the CD trace of the isolated NTR (Figure 4C). Qualitative CD spectral differences combined with secondary structure content predictions indicate that the N-terminal stretch spanning residue 1–111 is composed of disordered regions and secondary structures, both α-helical and β-strand (Figure 4C and Supplementary Figure S9) (40). The CD spectra of the N-terminal deletion and PAM2w mutants (79–287, 24–287, L15AW22A, L15A, W22A and W22F) however lose the deeper minima at ∼220–225 nm featured in the NTD wild-type spectrum, and display a similar curve shape as the La module but with significant less negative molar ellipticities (Figure 4C). This suggests that in the mutants the La module core structure is unaltered and that the N-terminal stretch contains random coil regions. It also maps secondary structure elements, possibly α-helical, within the first 24 residues of LARP4A NTD. Interestingly, such elements are destabilized by PAM2w amino acid substitutions, as inferred from the superimposable nature of the spectra of 24–287 and the four PAM2w mutant proteins. Notably, since PAM2w mutants are defective in their RNA binding capability, it appears that these transient secondary structure elements play a key role in RNA interaction. Furthermore, the RNA-binding incompetent fragments spanning 1–50 and 1–79 gave rise to CD profiles characteristic of random coil polypeptides (Figure 4C), supporting the view that a contiguous stretch of the N-terminal region of LARP4A appears necessary to retain the observed secondary structure composition, and that this is a prerequisite for RNA binding.

**Figure 6.**
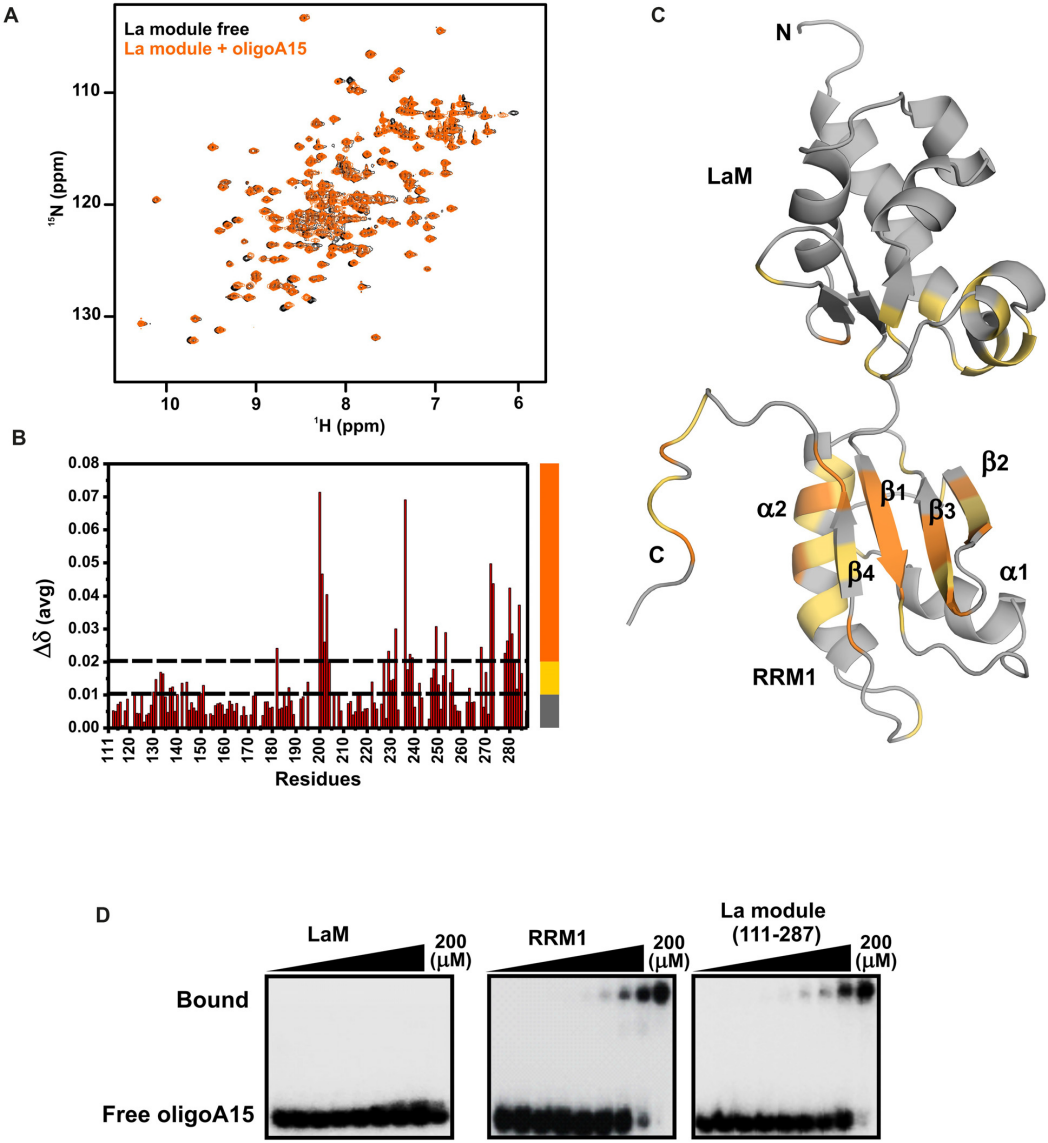
Analysis of the interaction between LARP4A and oligoA15 RNA. (**A**–**C**) NMR titration of the LARP4A La module with oligoA15. Panel (A) displays the overlay of the ^1^H-^15^N HSQC spectra of the La module in the apo and holo form (black and orange, respectively). Panel (B) shows protein chemical shift perturbations (Δδ_avg_) upon oligoA15 binding and (C) is the mapping of the Δδ_avg_ on a representative structure of the La module in cartoon mode representation. Two thresholds (represented with dotted lines in panel B) were considered in the analysis and colour coded on the structure (panel C) as follows: 0.01 < Δδ_avg_ ≤ 0.02 ppm in yellow and Δδ_avg_ > 0.02 in orange. Unaffected residues are depicted in grey. The secondary structure elements and the N- and C-termini are labelled on the structures. (**D**) The affinity of LARP4A LaM (left) and RRM1 (centre) and La module (right) for oligoA15 was assessed by EMSA. Protein concentrations of 0, 1.6, 3.1, 6.3, 12.5, 25, 50, 100 and 200 μM were used in the experiments. The RRM1 binds oligoA RNA with a similar affinity as the La module, indicating little (if any) involvement of LaM to binding.

The ^1^H-^15^N HSQC and ^1^H-^15^N TROSY-HSQC NMR spectra of a LARP4A NTD–oligoA15 RNA complex gave rise to extremely broadened signals in our experimental conditions (Supplementary Figure S12). Whereas this prevented a further analysis of the NTR region, we were able to follow the fate of the majority of the La-module signals, aided by NMR titration experiments conducted on the La module alone (Figure 6 and Supplementary Figure S12). Here, the addition of unlabelled oligoA15 to a sample of ^15^N-labelled La module revealed small chemical shift perturbations, indicative of protein-binding interfaces, that mapped almost exclusively to the RRM1 (Figure 6), in particular on the central β-sheet and helix α2. Although the exact interacting surface of the RRM1 remains to be delineated, these results propose a role for RRM1 in oligoA association, and this was corroborated by EMSA experiments performed with the RRM1 alone, which reproduced the oligoA15 binding profile of the La module (Figure 6D).

Thus, interestingly, the LaM remains largely unaffected. We noted that LARP4 family members that evolutionarily acquired the PAM2 motif were characterized by a lack of conservation for some of the six salient residues identified in La (11). To examine whether this could account for the lack of involvement in oligoA binding, C130 and M160 were replaced with the otherwise conserved Y (in hLa, this would be position Y24) and F (numbering 55 in hLa), respectively. The double C130YM160F mutant however failed to bind oligoA any tighter than the wild-type La module (Supplementary Figure S13).

## DISCUSSION

In this work, we have used biochemical, biophysical and structural methods to analyse the interactions between LARP4A and two of its direct partners, oligoA and PABP. We report here the discovery and characterization of an unprecedented RNA-binding mechanism by LARP4 mediated by intrinsically disordered regions and structured elements that do not contain recognizable RNA-binding motifs. The La module of LARP4A, initially anticipated to be the main locus of RNA binding, was instead found to play a minor role. By using the N-terminal sequences to bind oligoA, LARP4A engages its PAM2w motif in this recognition. This motif, traditionally used as a PABP interaction surface by a multitude of proteins with roles in translation, was here for the first time found to mediate direct poly(A) RNA interaction. This thereby unveiled a functionally important interplay between LARP4A partners, in that LARP4A binding to oligoA RNA is mutually exclusive with PABP MLLE.

### A novel RNA-binding mode for LARP4A

**Figure 7.**
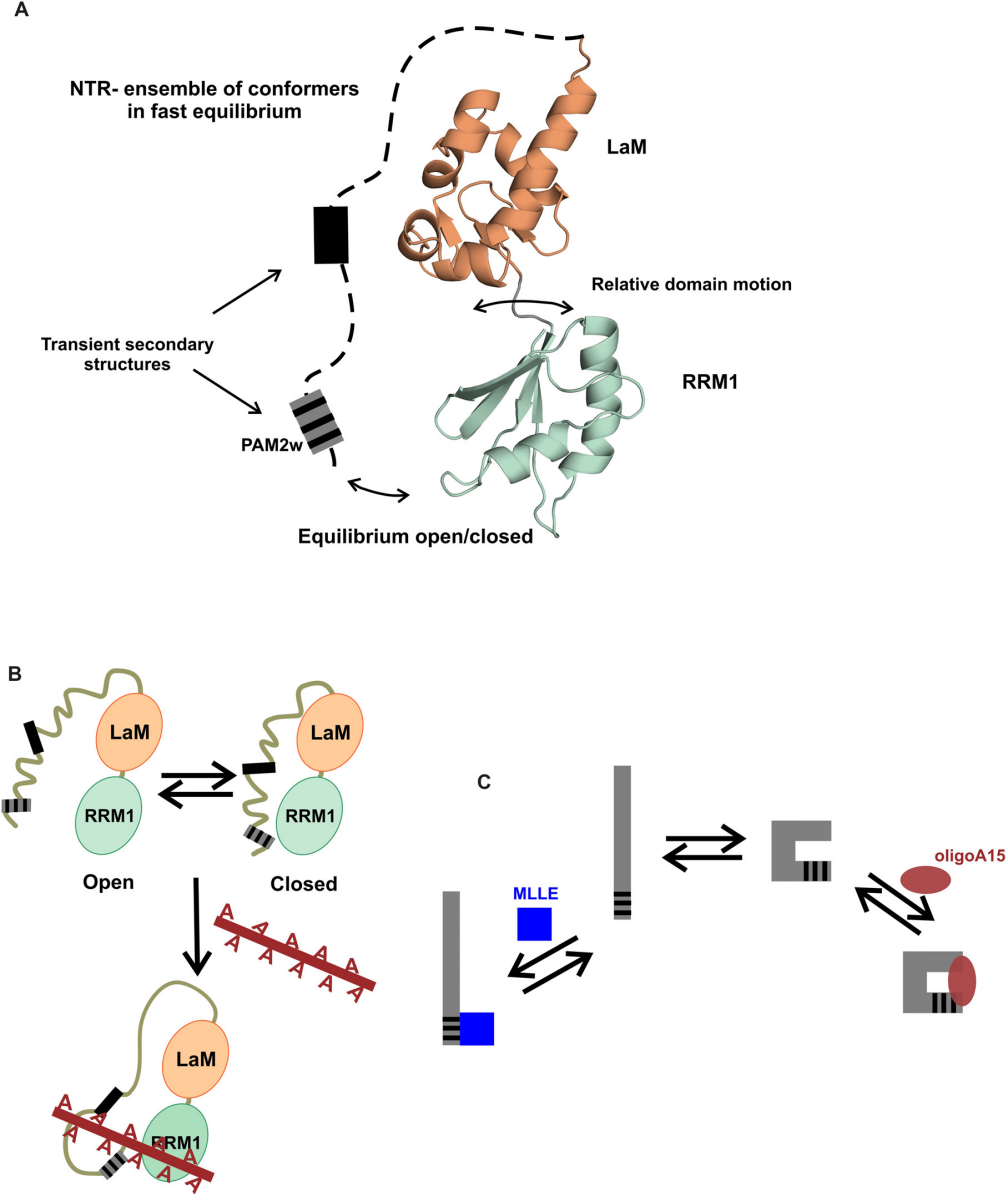
A structural model for LARP4A NTD and its interaction with oligoA RNA. (**A**) A structural model of LARP4A NTD showing a representative structure of the La module flanked by a disordered N-terminal arm. The NTD is made up by flexible moieties: the LaM and RRM do not adopt a rigid relative orientation and the NTR undergoes motions on the picosecond to the nanoscale time scale. The NTD ensemble of interconverting conformers also includes a millisecond timescale equilibrium between open and closed forms. The NTR is arbitrarily represented with a dotted line. Grey boxes in the NTR loosely map regions with secondary structure propensities, evinced from biophysical, bioinformatics and mutagenesis studies (see text). The grey and black-striped box represents the PAM2w motif. (**B**) Model of LARP4A NTD–oligoA interaction. Regions in the NTR within the first 24 residues, and between aa 24 and 79, have been implicated in RNA interaction, as well as the RRM1. In this model, other NTR regions are shown not in contact with the RNA, but this is an assumption to be confirmed experimentally. (**C**) Schematic representation of the proposed NTR conformational selection. The NTR exists as many interconverting conformers in equilibrium, where the binding competent conformations are pre-existing in solution prior to the binding of the ligands, either MLLE domain or oligoA15 RNA.

Contrary to initial hypotheses, the entire NTD of LARP4A was found to be the minimal oligoA15 RNA-binding region, with the N-terminal 111 residues, the NTR, acting as the main determinant for oligoA recognition, and the La module, in particular the RRM1, contributing to a lesser extent to the interaction. The NTD does not fold into a defined tertiary structure: it is composed of two stable independently folded domains, the LaM and the RRM1 that do not adopt a fixed orientation with one another, and are preceded by the NTR, which exhibits considerable dynamics on the pico to nanosecond timescale and contains transient secondary structure elements (Figure 7). Our NMR analyses also suggest that LARP4A NTD populates interconverting closed/open conformations in equilibrium, regulated by transient interactions between the La module and the N-terminal regions, a motion on the millisecond timescale. In its semi-disordered state, the NTD can nonetheless function as RNA-binding platform. Notably, primary structure conservation of NTR regions across LARP4A proteins is very high, indicating that the inherent conformational flexibility finely tuned by the protein sequence is evolutionarily conserved (55), and likely to reflect the functional constraints imposed by the interaction with RNA and other ligands.

The finding that the N-terminal regions of LARP4A are prevalent partners in oligoA interactions was unexpected: the NTR defies convention by lacking discernible RNA-binding motifs or domains, encompasses a well-characterized protein–protein interaction site (the PAM2w motif) and contains semi-disordered flexible regions, which have only recently begun to emerge as main actors in RNA-binding activities (55,56). Intriguingly though, LARP4A NTR does not show resemblance to any known intrinsically disordered RNA-binding motifs or any other recently reported by proteome-wide scale studies, the majority of which included RG- and RS-repeats, (G/S)Y(G/S) motifs and K- or R-rich basic patches (51,56). The NTR conversely is predominantly acidic, with high proportion of Glu, Ser, Thr and Gly. This finding therefore expands the current knowledge of the ‘dark proteome’ that may be directly involved in RNA biology processes (57), as well as providing new insights into the ever-growing heterogeneity of protein–RNA interactions.

Intrinsically, disordered regions of proteins have been documented to participate in a plethora of cellular and molecular recognition processes (58,59). The major advantages of conformational flexibility lie with the potential to mould into a particular binding surface and/or with the ability to support multifunctional regions, capable of targeting different binding partners in a context-dependent manner (58,60). Based on the data presented here, we hypothesize that the ensemble of different conformational substates representing the NTD may have evolved to enable LARP4A to interact with multiple ligands, in particular oligoA RNA and PABP MLLE, and perhaps others yet to be identified. Our CD and mutagenic analyses implicate the secondary structure propensity of the NTR as a determinant for oligoA binding, given that N-terminal truncation and PAM2w mutants that remove or destabilize preformed local α and/or β structure greatly decrease the affinity for this ligand, presumably by disfavouring the NTD conformation(s) competent for RNA association (58). This would imply that LARP4A NTD achieves specific RNA recognition mainly through a conformer selection mechanism (60), although this remains to be demonstrated.

If RNA recognition relies on the population of secondary structure present in the unbound state of the NTR, the interaction of its PAM2w motif to MLLE does not, and may well be disfavoured by it, in view of the fact that all the PAM2 motifs known thus far have been mapped to unstructured regions of proteins (24,61). LARP4A W22A and W22F mutants with diminished secondary structure propensity in the NTR can still associate with the MLLE domain whilst being RNA-binding deficient, reinforcing the idea that structural polymorphism and conformational plasticity of LARP4A NTR endow it with the ability to serve as interacting region for (at least) two distinct partners. In other words, conformational fluctuations of the NTR transiently expose the PAM2w motif in a largely unstructured conformation, enabling it to interact with PABPC1, and transiently populate an ‘active’ substate competent for RNA interaction, with secondary structures that select the RNA partner (Figure 7). This molecular switch model concurs with the mutually exclusive oligoA/MLLE binding our findings have unveiled, although the full structural bases for this competition mechanism are yet to be understood.

From our investigations, we conclude that binding of LARP4A to RNA involves a complex dynamic of modular domains and unstructured regions whose synergic and dynamic interplay is likely to play key roles in RNA-binding selection. Although a detailed picture awaits the characterization at atomic level of a LARP4A–oligoA complex, taken together our data allow the formulation of a first molecular model for oligoA interaction, in which oligoA spans from a region in the NTR across to make some contact with the RRM1 (Figure 7). In the NTR, the first 24 residues are likely implicated, although other regions are not excluded, for example the stretch around residue 60 (see above). It may also be possible to envisage that the open/closed conformation can be switched by the presence of RNA and protein partners that bind to the NTR, but this remains to be demonstrated.

Finally, it is noteworthy that disordered regions of proteins have been shown to form a fluid matrix in membraneless organelles that contain RNA and proteins and are thought to be fulcrums of RNA processing, degradation, transcription and translation (62,63). It may therefore be interesting to investigate whether LARP4A plays a role in regulating phase separation transitions for cytoplasmic organelles, such as P bodies and stress granules. Of note, LARP4A was found to localize to stress granules following arsenite treatment (1,64).

### A first unconventional La Module in LARP4A?

The La module is the central RNA-binding unit found conserved in all LARPs (2), composed of a LaM paired with an adjacent RRM1. Previous studies on La revealed that RNA target recognition is achieved via conformational realignment of the LaM and RRM1 with respect to one another, aided by the interdomain linker, which secures the position and the specific contacts of the cognate RNA in the binding crevice of the protein (13). Despite RNA substrate variability, the specific mode of recognition of other La modules characterized to date, *i.e*. human LARP7 and LARP6, follows a common theme, invariably involving cooperation of the LaM, RRM1 and linker, and this was presumed to be a convergent and distinguishing trait across the LARP superfamily (2,16,17). The findings reported here however challenge the notion that the La module in its entirety is at all times partaking in RNA binding, since the La module of LARP4A contributes to oligoA association only through the RRM1. The LaM of LARP4A appears peripheral to this process, despite possessing a similar winged-helix scaffold to La, LARP7 and LARP6, and a comparable size and electrostatic potential in and around the hydrophobic crevice (17) (Supplementary Figure S3). An initial hypothesis that the lower degree of conservation of the six key residues may have rendered the hydrophobic pocket of LARP4A LaM non-functional that was not backed up by experimental evidence. Notably, a unique feature of LARP4A LaM revealed by our structural work is the loss of the wing2, prompting suggestions that this structural perturbation could be responsible for the reported behavioural variation. In support of this, the missing LaM C-terminal wing coupled with a short interdomain linker imposes an overall divergent architecture of the La module of LARP4A from previously studied systems (2). Whether this new tandem domain arrangement prevents the La module of LARP4A from using the cooperative power of the LaM and RRM1 for RNA recognition remains to be clarified. Of note, in a previous work LARP4A La module was reported to bind to oligoA15 in the high nanomolar affinity (1) by isothermal titration calorimetry (ITC). Follow-on analysis, however, revealed that ITC measurements were hampered by protein aggregation occurring overtime upon RNA titration (not shown).

Whereas the lack of the observed LaM/RRM1 synergism concurs with the modest participation of LARP4A La module to oligoA RNA binding, it cannot be excluded that La module could suffice for selection of other RNA substrates. Notably, new RNA targets of LARP4A are emerging, e.g. recently LARP4A was found to stabilize the miR-210 locus, mostly likely by binding to pre-miR210 (4). Such a dual mode for RNA recognition would not be unprecedented: the La protein for instance was found to engage its La module to bind specifically to short 3′ oligoU sequences, whereas cooperation of the La module with the C-terminal RRM (RRM2) enables the association of La with the stem–loop IV of hepatitis C virus (65). Beyond LARPs, the modular architecture featured in the majority of RNA-binding proteins results in increased versatility, allowing for multiple target selection and expanding the functional repertoire of these proteins (66,67).

### A new function for the PAM2w motif and implications for LARP4A, PABP and RNA interplay

PAM2 motifs are functionally important segments that mediate the interaction of many proteins with PABP, in particular its C-terminal MLLE domain (24). These PAM2-containing proteins include factors with varied roles in either RNA metabolism or translational regulatory pathways. Here, we have discovered for the first time a PAM2 motif partaking in RNA association, whilst retaining canonical MLLE domain interaction. To our knowledge, this is the first example of its kind, thereby ascribing a new function (RNA binding) to this protein–protein interaction motif. Whether such a dual role is a sole prerogative of the LARP4A PAM2w variant or a more universal feature of PAM2 motifs remains to be seen. Notably, our data clearly indicate that LARP4A PAM2w alone is not sufficient to bind oligoA, but it is necessary within a defined structural/sequence context of the NTR. Furthermore, the finding that the W22F mutation is detrimental for oligoA binding confers a key role to the variant tryptophan residue W22 in RNA interaction, and provides a mechanistic explanation for this divergent position, which is an absolutely conserved phenylalanine in all the other PAM2 motifs known to date (11,19). As LARP4B proteins also contain a variant PAM2w motif, parallels could be drawn between the LARP4A and LARP4B families, although it is noteworthy that their NTR sequences differ somewhat especially at the N-terminus. Beyond LARP4B, it may be interesting to survey the RNA-binding properties of PAM2 motif-containing regions of other factors, in particular LARP6 proteins that, analogously to the LARP4 family, have acquired the PAM2 following neofunctionalization of the LaM (e.g. plant LARP6B and LARP6C) (11). Although in the LARP6 family, the PAM2 sequence is canonical, one can still speculate that such evolutionary process in the LARPs may have offered extra RNA-binding sites and/or caused changes in canonical sites (i.e. the LaM), thereby contributing to RNA target selectivity. This hypothesis remains to be tested.

**Figure 8.**
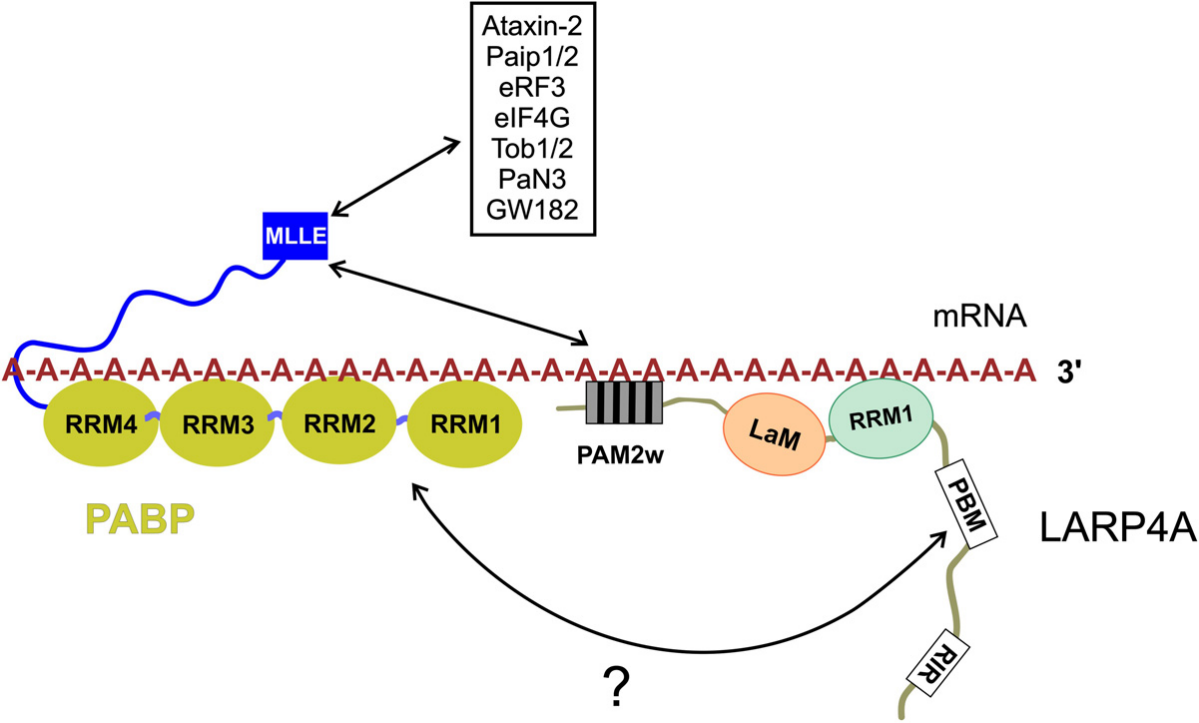
Working model of the interplay between PABP, LARP4A and mRNA. A network of interactions between PABP, LARP4A, polyA RNA and other PAM2-containing factors that bind PABP through the MLLE domain is shown. PolyA RNA competes for LARP4A NTD interaction with the MLLE domain, possibly regulating LARP4A polyA mRNA coverage and the recruitment of other PAM2-containing factors to the 3′UTR.

The duality of LARP4A PAM2w motif unveils a tantalizing functional interplay between protein–RNA and protein–protein interaction, in that LARP4A binding to oligoA is incompatible with concomitant MLLE interaction. The biological relevance of the mutual exclusivity warrants consideration. Not only the PAM2w confers LARP4A the ability to bind oligoA, but, notably, oligoA binding to LARP4A provides the means to regulate reversibly its PABP MLLE interaction. This is particularly interesting, given that to date the molecular mechanism(s) that control association and dissociation of the many PAM2-containing proteins to PABP in response to different biological processes and stimuli are unclear, albeit phosphorylation of PAM2-surrounding residues was suggested as a possible dissociation signal (61). Our study therefore may provide a possible molecular mechanism for LARP4A, whereby oligoA binding is controlling dissociation of LARP4A from MLLE domain. We note that the affinity of LARP4A for RNA is ∼10-fold higher than for MLLE. We thus propose that by direct competition oligoA RNA would prevent the interaction between LARP4A NTD and the MLLE domain, driving the handover of MLLE from LARP4A to other PAM2-motif containing proteins (Figure 8). Such model would not necessarily rule out the formation of a ternary complex between LARP4A, polyA RNA and PABP, mediated by the association of PBM with yet unidentified surfaces on PABP, in line with the bipartite mode of interaction proposed for LARP4A and PABP (1,2). Further investigations will be required to validate the proposed hypotheses, possibly involving full-length proteins to assess the relative importance and any cross-talk between the two LARP4A–PABP interacting regions.

The interplay between polyA, PABP MLLE and LARP4A may constitute a hitherto unknown mechanism for regulating mRNA translation, stability and polyA lengths, and could serve as a feedback mechanism to control PABP activity in the cell and mRNA turnover (1,3,20,68). The observation that LARP4A PAM2w mutant is defective in polyA lengthening (3) could perhaps be explained by its low affinity for polyA, as LARP4A association with the polyA tail could be intuitively considered as protecting mRNA against degradation by exonucleases, although this requires further experimentation. The LARP4A–MLLE and LARP4A–oligoA mutual exclusive complexes could be components of different mRNA processes (e.g. acting on distinct mRNAs) or alternatively be remodelled assemblies at different time points in the translation control of the same mRNA.

In conclusion, our study reveals an unprecedented mode of RNA recognition by LARP4A using unorthodox motifs. The insight into structural and functional effects of LARP4A represents a significant advance towards mechanistic understanding of the role of LARP4A in enhancing mRNA stabilization. The findings observed here for LARP4A suggest that PAM2w motif protein may not only mediate interaction with PABP, but also have more general and novel roles in the regulation of protein function in translation.

## DATA AVAILABILITY

The atomic coordinates for the NMR ensembles of LARP4A La module are deposited in the Protein Data Bank under accession number 6I9B. The chemical shift assignments have been deposited in the Biological Magnetic Resonance Data Bank (26).

## Supplementary Material

Supplementary Data
