# Supplementary material for: LARP4A recognizes polyA RNA via a novel binding mechanism mediated by disordered regions and involving the PAM2w motif, revealing interplay between PABP, LARP4A and mRNA
Source: Nucleic Acids Res. 2019 Mar 1;47(8):4272–91. doi: 10.1093/nar/gkz144 (PMC6486636; doi:10.1093/nar/gkz144)
Supplement: Supplementary Data [file gkz144_supplemental_file.pdf]

Supplementary information

LARP4A recognises polyA RNA via a novel binding mechanism mediated by disordered regions and involving the PAM2w motif, revealing interplay between PABP, LARP4A and mRNA

Isabel Cruz-Gallardo[1], Luigi Martino[1], Geoff Kelly[2], R. Andrew Atkinson[1,3], Roberta Trotta[1], Stefano De Tito[1], Pierre Coleman[1], Zainab Ahdash[4], Yifei Gu[1], Tam T.T. Bui[1,3] and Maria R Conte[1,3*]

[1]Randall Centre for Cell and Molecular Biophysics, King's College London, London SE1 1UL, UK; [2]MRC Biomedical NMR Centre, The Francis Crick Institute, London NW1 1AT, UK; [3]Centre for Biomolecular Spectroscopy, King's College London, London SE1 1UL, UK; [4]Department of Chemistry, King's College London, London SE1 1DB, UK

* To whom correspondence should be addressed: Tel. +44 207 8486194; Email:sasi.conte@kcl.ac.uk

Present address: Isabel Cruz-Gallardo, Department of Chemistry, King's College London, London SE1 1DB, UK; Luigi Martino, The Francis Crick Institute, 1 Midland Road, London NW1 1AT, UK; UK, Stefano De Tito, Institute of Protein Biochemistry, National Research Council, Via Pietro Castellino 111, 80131 Naples, Italy; Pierre Coleman, School of Cardiovascular Medicine & Science, The Rayne Institute, King's College London, London SE1 7EH, UK

This document contains Supplementary Figures S1-S13

Figure S1

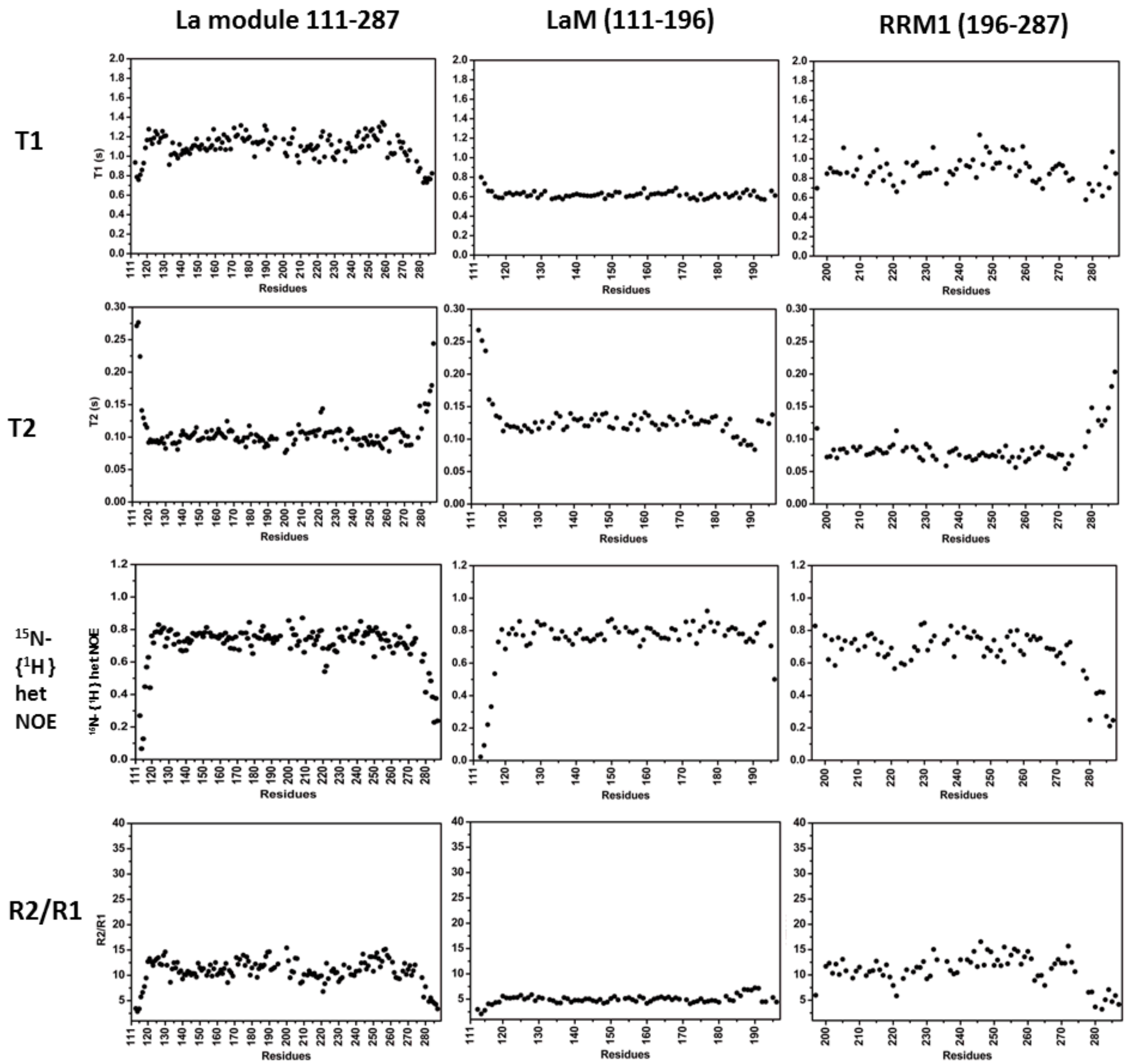

Figure S1. NMR backbone relaxation analysis. T1, T2 and ^{15}N-{^{1}H} NOE values were measured at 700MHz and 298 K for LARP4A La module (*left*), LaM (*centre*) and RRM1 (*right*). A plot of R2/R1 values is also shown for the 3 proteins.

Figure S2

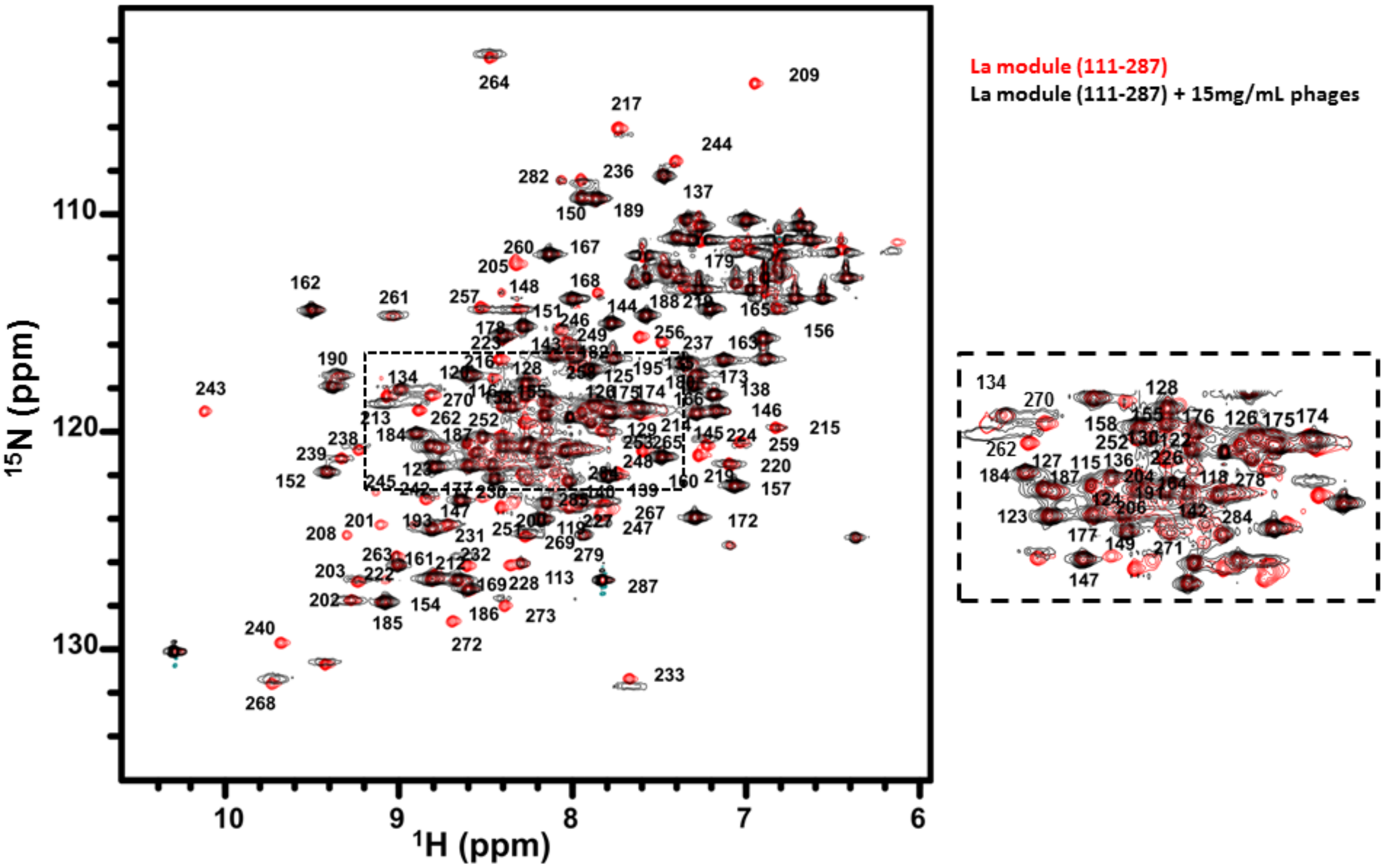

Figure S2. Analysis of LARP4A La module in liquid crystalline media. Overlay of ^{1}H-^{15}N HSQC spectra of LARP4A La module in buffer (red) and in the presence of 15 mg/mL Pf1 phages (black). The residues of the RRM1 either disappear or are significantly broadened. A closer view of the central part of the spectra is shown for clarity.

Figure S3

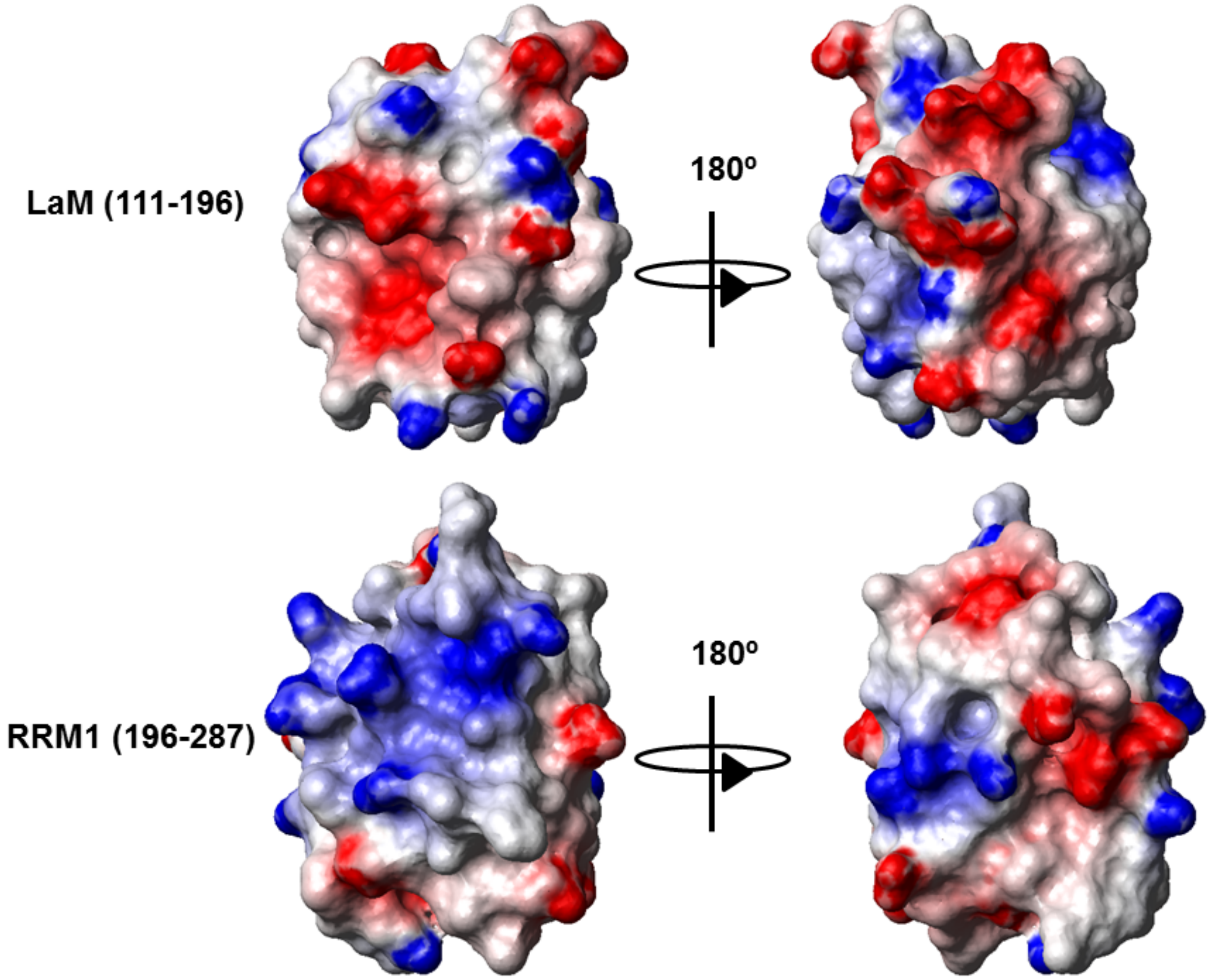

Figure S3. Electrostatic surface potential of LARP4A LaM and RRM1. LARP4A LaM (top) and RRM1 (bottom) are shown on the left in the same orientations than Figure 1**b** and **c**. Structures on the right show a 180° rotation. Red and Blue highlight acidic and basic regions, respectively. The figures were created using MOLMOL (1) (see Methods).

Figure S4

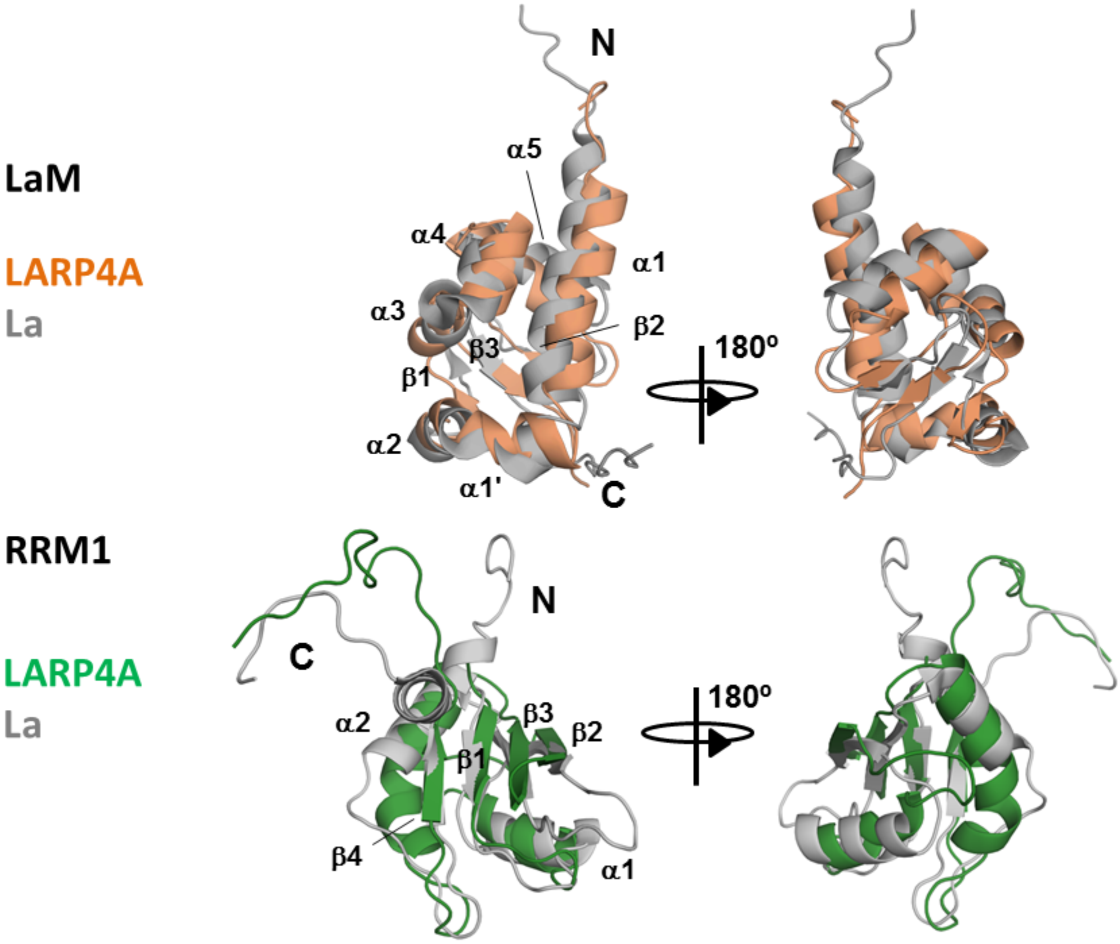

Figure S4. Structural comparison between the La module of human LARP4A and human La. Structural overlays of the LaM (top) and RRM1 (bottom) of human La and LARP4A proteins shown on the left in the same orientations than Figure 1**b** and **c**. The La proteins are in grey and LARP4A in orange (LaM) and green (RRM1). The N and C-termini, α-helices and β-strands are indicated. Figures were prepared with PyMOL (https://pymol.org/2/).

Figure S5

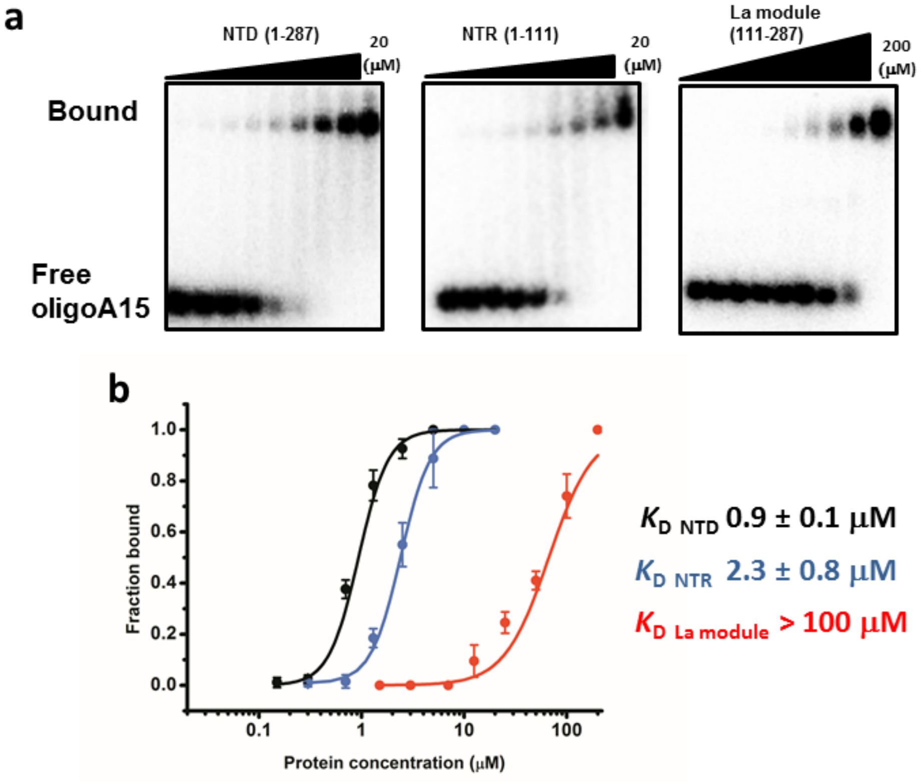

Figure S5. EMSA binding assays of LARP4A N-terminal domain (NTD), N-terminal region (NTR) and La module with ^{32}P-oligoA15 in absence of competitor tRNA mix. a. Representative autoradiograms are shown for NTD (*left*, top), NTR (*centre*, top) and La module (*right*, top). For LARP4A NTD and NTR, protein concentrations of 0, 0.15, 0.3, 0.7, 1.3, 2.5, 5, 10 and 20 µM were used; for the La module the concentrations were 0, 1.6, 3.1, 6.3, 12.5, 25, 50, 100 and 200 µM. Bound and free RNA populations are labeled. **b.** Binding curves showing fractions of protein-bound RNA plotted as a function of protein concentration and the fitting of the data. Average values for K_D (dissociation constant) and standard deviations reported were calculated from at least 3 biological repeats. The dissociation constants for LARP4A NTD and NTR were 2-3 fold higher in presence of competitor tRNA (Figure 2), supporting overall binding specificity. The behaviour of the La module was also very similar in the two different conditions, but any quantification was hindered by the weak nature of the interaction.

Figure S6

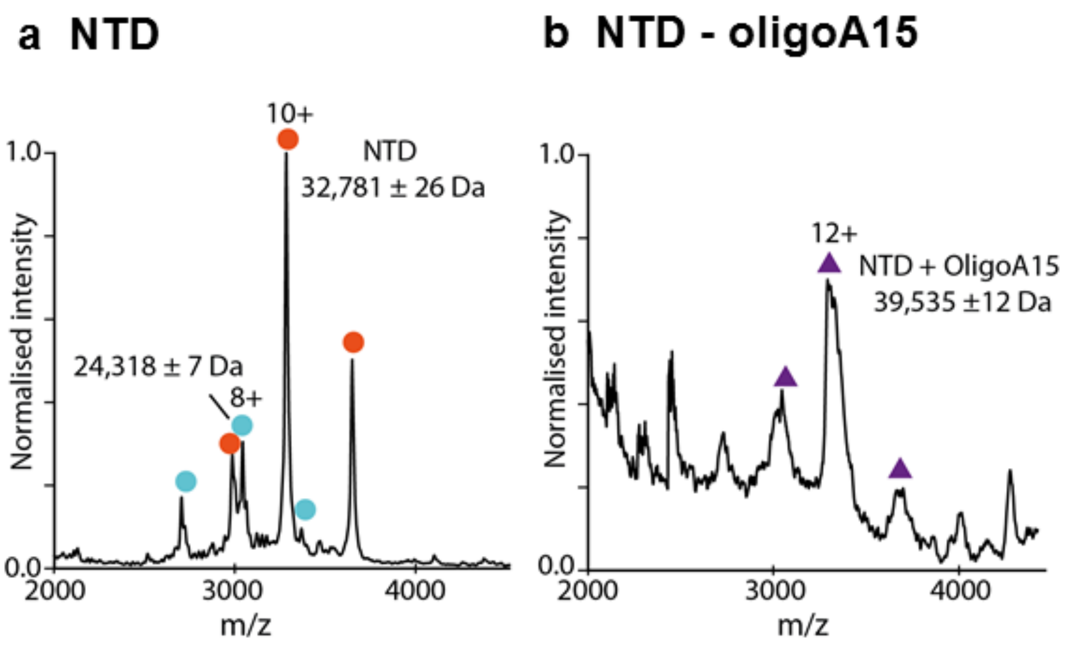

Figure S6. **Stoichiometry determination of LARP4A NTD-oligoA15 complex.** Native MS was performed LARP4A NTD in **a.** the apo form and **b**. bound to oligoA15. The species at 24,318 Da in panel **a** correspond to a degradation product of the NTD generated during the experiment.

Figure S7

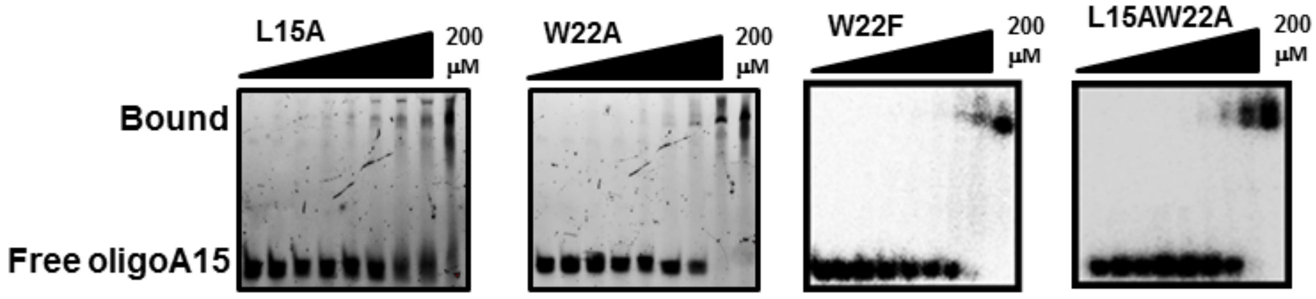

Figure S7. Mutations within the PAM2w motif affect LARP4A NTD-oligoA association. The interaction of LARP4A PAM2w mutants with oligoA15 was assessed by EMSA. L15A and W22A mutants were tested with 5’FAM oligoA20, whereas W22F and L15AW22A mutants were tested with ^{32}P-oligoA15, as described in the Methods. Free and Bound RNA species are labelled. The experiments were conducted with protein concentrations of 0, 1.6, 3.1, 6.3, 12.5, 25, 50, 100 and 200 μM.

Figure S8

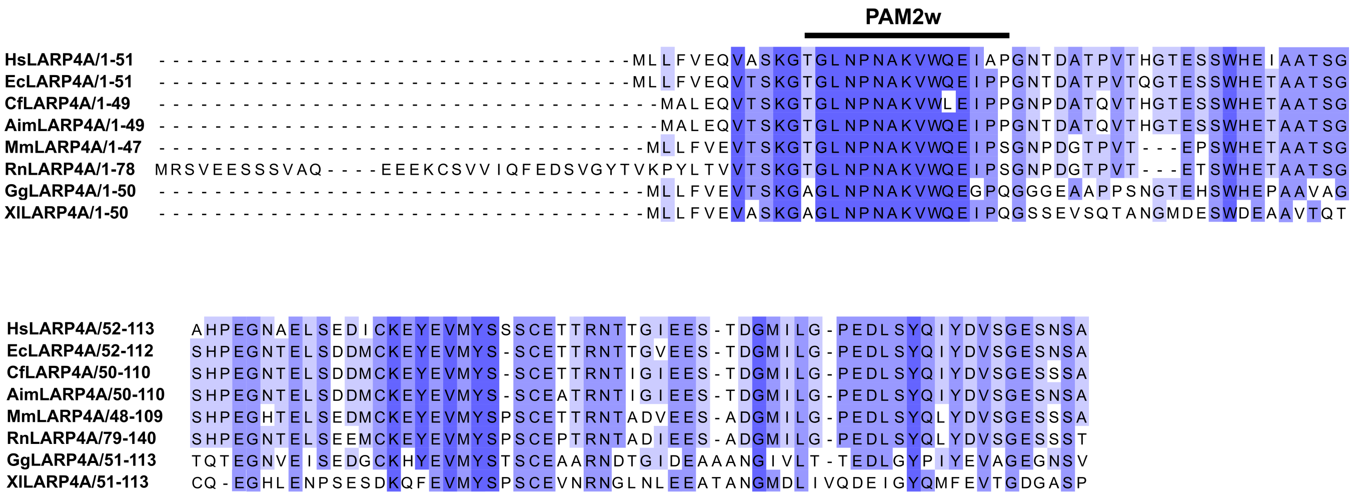

Figure S8. The NTR is highly conserved in tetrapod LARP4A proteins. The sequence of human LARP4A NTR was aligned with proteins of different species using Clustal Omega in Uniprot website (http://www.uniprot.org/align/). The alignments were edited and analysed with Jalview software (2). Residues were coloured in a blue scale according to the extent of conservation. The species codes are: (Hs) *Homo sapiens*; (Ec) *Equus caballus*; (Cf) *Canis familiaris*; (Aim) *Ailuropoda melanoleuca*; (Mm) *Mus musculus*; (Rn) *Rattus norvegicus*; (Gg) *Gallus gallus* and (Xl) *Xenopus laevis*.

Figure S9

a

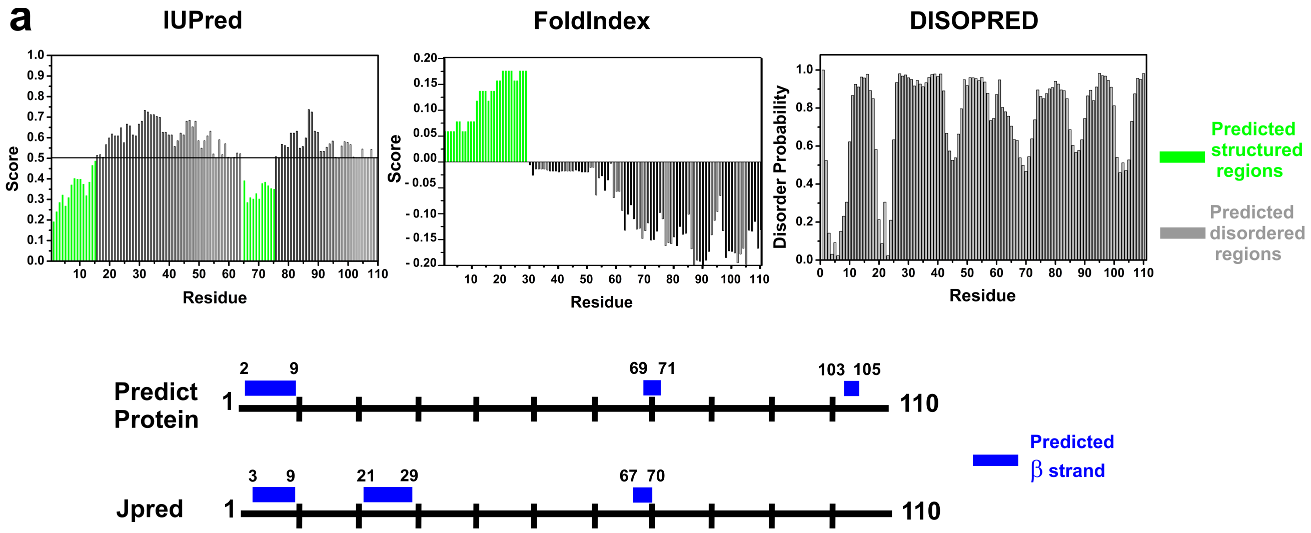

b

Protein	α	β	turn	other	Number of residues
NTD (1-287)	26.1% (74 res)	12.3% (35 res)	14.7% (42 res)	47.0% (136 res)	287
La-module (111-287)	27.4% (49 res)	11.5% (21 res)	14.5% (26 res)	46.0% (83 res)	179
NTR (1-111)	14.3% (16 res)	19.5% (22 res)	14.6% (16 res)	51.7% (57 res)	111
L15AW22A	18.9% (54 res)	15.1% (43 res)	15.7% (45 res)	50.2% (145 res)	287
W22F	20.0% (58 res)	17.1% (49 res)	15.8% (45 res)	47.1% (135 res)	287
24-287	17.7% (47 res)	16.5% (43 res)	16.2% (43 res)	49.5% (131 res)	264
79-287	22.7% (47 res)	17.8% (37 res)	14.9% (31 res)	44.6% (94 res)	209

Figure S9. **Secondary structure and disorder analysis for LARP4A NTD and mutants**. Panel **a** reports secondary structure and disorder predictions for LARP4A NTR (residues 1-110), derived from web servers IUPred (3), FoldIndex (4), DISOPRED (5), JPred (6) and PredictProtein (7) (see Methods). Predicted ordered and disordered regions are showed in green and dark grey respectively. IUPred calculates a probability score which ranges from 0 (maximum order) to 1 (maximum disorder), with scores above 0.5 ascribed to disorder. The Foldindex score indicates a propensity to fold, with values above 0 for folded regions and values below 0 for disordered regions. DISOPRED provides a probability of disorder ranging from 0 to 1, where 1 indicates maximum disorder. Predicted secondary structures from JPred and PredictProtein are showed in blue in a schematic representation of protein sequence, and annotated with residue numbers. The remaining parts were predicted not to contain secondary structure elements. **b.** Estimated secondary structure content from circular dichroism (CD) data using BeStSel (8) (see Methods) for various LARP4A mutants. Percentage and corresponding residue length are given for each protein.

Figure S10

a

Domain	Chemical shifts variations		Unperturbed residues	Unclear		
	$0.015 \le \Delta\delta_{avg} \le 0.03$	$\Delta\delta_{avg} > 0.03$		Spectral overlap	Unassigned residues	Prolines
**LaM**	114, 115, 116, 122, 123, 125, 127, 130, 131, 134, 135, 136, 137, 139, 144, 149, 156, 158, 161,167, 168,169, 176, 177, 179, 180, 182, 183, 184, 185, 186, 190, 193	113, 117, 124, 140, 162	120, 121, 133, 138, 145, 146, 147, 148, 150, 151, 152, 154, 155, 157,159, 160, 163, 165, 171, 172, 173, 174, 175, 178, 188, 189, 192	118, 119, 126, 128, 129, 141, 142, 143, 164, 166, 187, 191, 195	111,112,132,196, 197,198, 199	153, 170, 181, 194
**RRM1**	202, 203, 205, 208, 209, 213, 221, 230, 231, 232, 233, 238, 240, 243, 244, 248, 252, 253, 268, 270, 273, 280, 282, 287	201, 237, 242, 272	210, 212, 214, 215, 216, 217, 218, 219, 220, 222, 223, 224, 228, 236, 239, 245, 248, 251, 255, 256, 257, 258, 259, 261, 262, 263, 264, 265, 279	200, 204, 206, 226, 241, 246, 247, 249, 250, 254, 260, 267, 269, 271, 274, 278, 283, 284, 285, 286	234, 235, 275, 276, 277, 281	207, 211, 225, 266

b

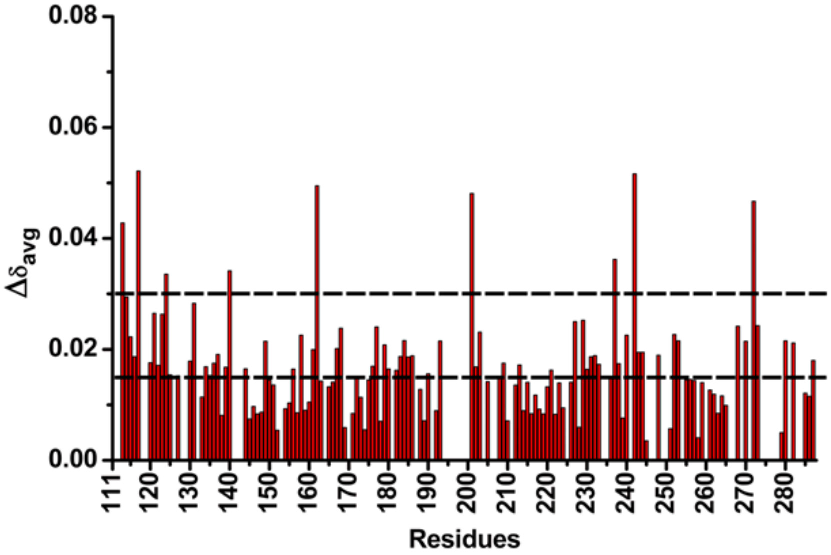

Figure S10. Amide signals of LARP4A La module experience minor chemical shift variations in the context the entire NTD. a Table showing an analysis of ^{15}N and $^{1}H_N$ chemical shift perturbations of LARP4A La module residues in the context of the entire NTD. $\Delta\delta_{avg}$ was calculated as $\{0.5\ [\Delta\delta(^{1}H_N)^2 + 0.2\ \Delta\delta(^{15}N)]^2\}^{1/2}$. Chemical shift perturbations are clustered in groups according to the extent of the perturbation. **b** Plot of the chemical shift variations detailed in **a**. The thresholds represented with dotted lines in **b** correspond to the $\Delta\delta_{avg}$ group classification used in **a**.

Figure S11

a

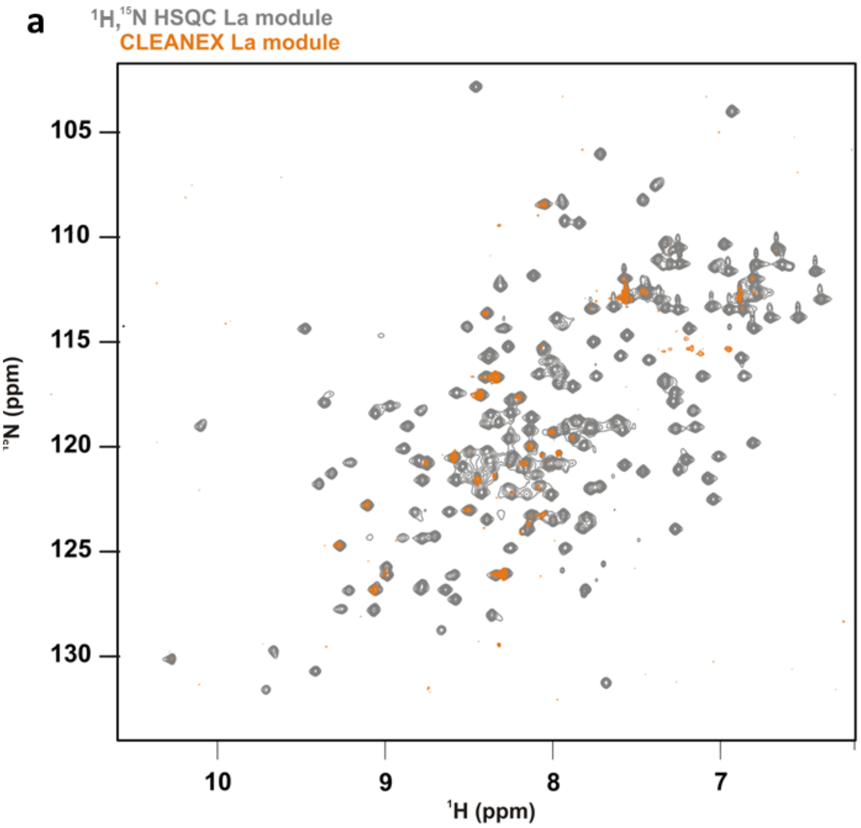

Residues	La module	NTD	CSP (ppm)
113	E	R	0.035
114	E	P	0.015
115	E	R	0.020
116	E	R	0.016
117	E	P	0.031
148	E	P	0
161	E	P	0.009
179	E	P	0.008
187	E	P	unclear
208	E	P	0.011
222	E	P	0
245	E	P	0
246	E	unclear	unclear
263	E	P	0
280	E	P	0.008
282	E	P	0.012
283	E	unclear	unclear
285	E	unclear	unclear
286	E	unclear	unclear

b

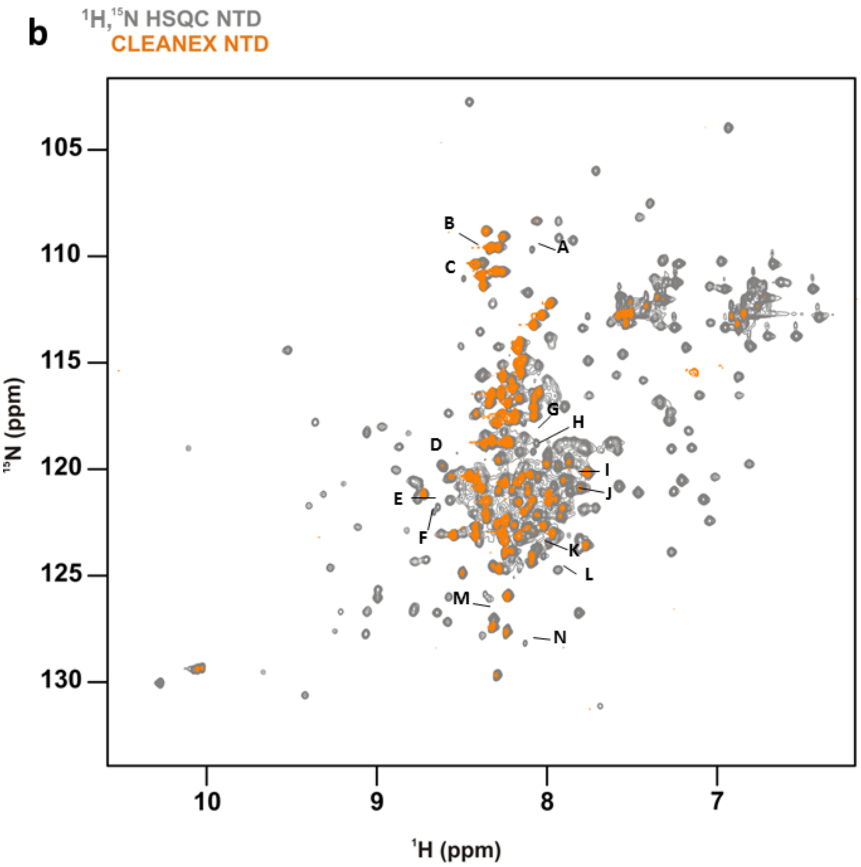

Protected NTR resonances	^{1}H (ppm)	^{15}N (ppm)
NTR-A	8.1	109.8
NTR-B	8.4	110.4
NTR-C	8.5	111.1
NTR-D	8.6	119.9
NTR-E	8.6	121.9
NTR-F	8.7	122.1
NTR-G	8.1	118.9
NTR-H	8.1	119.3
NTR-I	7.9	120.6
NTR-J	7.9	121.1
NTR-K	8.1	123.8
NTR-L	8.0	124.2
NTR-M	8.3	127.1
NTR-N	8.1	128.3

Figure S11. CLEANEX experiments indicate transient contacts between LARP4A La module and NTR. CLEANEX-PM experiments (9) were conducted on LARP4A La module in isolation and in the context of NTD. **A**. *Left,* CLEANEX spectrum of the La module in isolation recorded with 100 ms mixing time (in orange) overlaid over the control ^{1}H-^{15}N HSQC (in grey). *Right*, comparison of La module residues that are solvent-exposed in the La module in isolation but protected in the NTD. E indicates solved exchange, R indicates reduced solvent exchange rate and P indicates protection from exchange. Their chemical shift perturbations (CSP) in the context of the NTD are also reported, calculated as $\{0.5\ [\Delta\delta(^{1}H_N)^2 + 0.2\ \Delta\delta(^{15}N)]^2\}^{1/2}$. **b**. *Left,* CLEANEX spectrum of LARP4A NTD recorded with 100 ms mixing time (in orange) overlaid over the control ^{1}H-^{15}N HSQC (in grey). *Right*, list of non-assigned residues belonging to the NTR which are not exchanging with the solvent in the timescale of our experiments. The residues have been arbitrarily named as "*NTR-letter*" from A-N and labelled in the spectrum on the left.

Figure S12

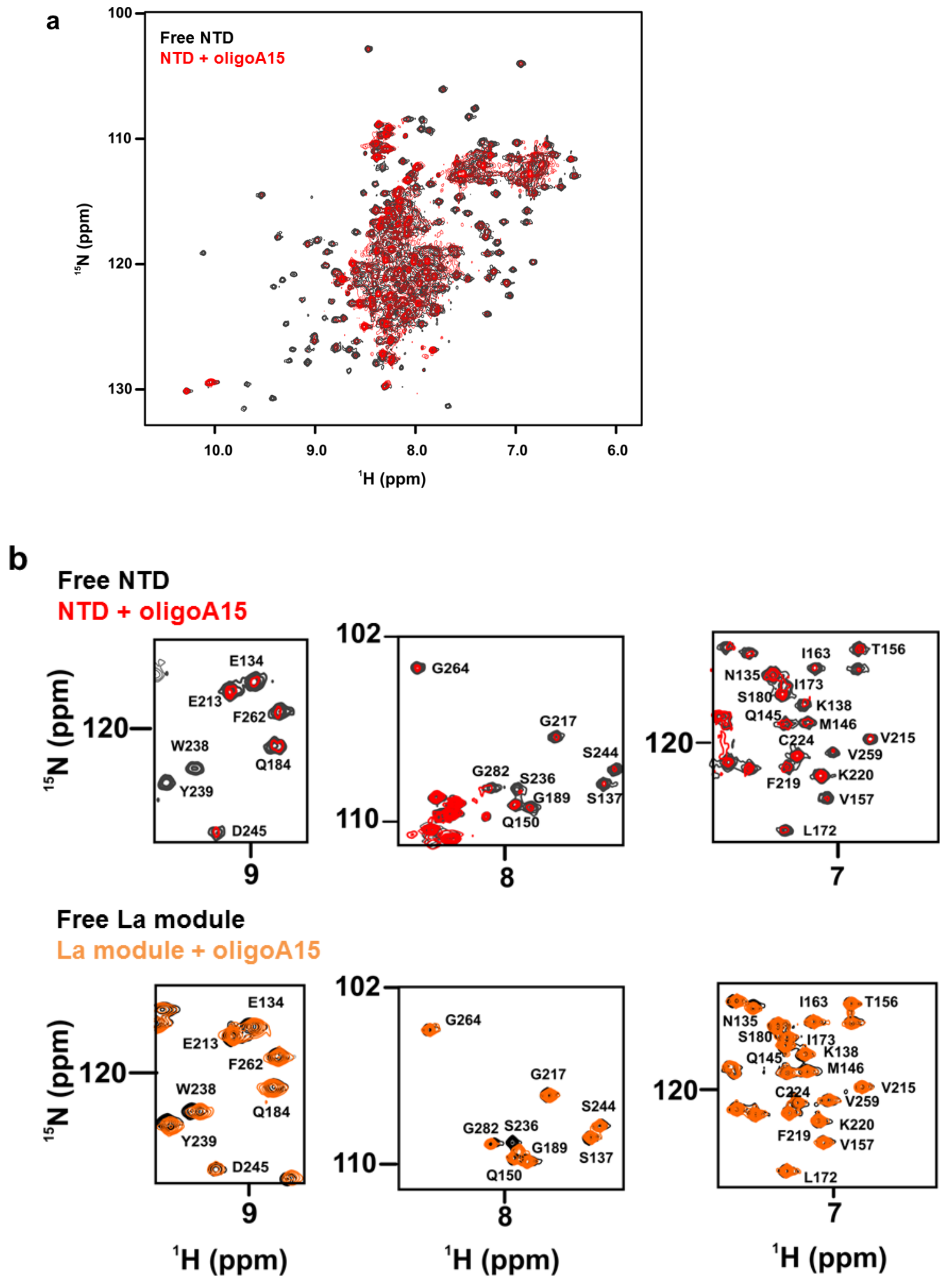

Figure S12. NMR analysis of LARP4A NTD-oligoA interaction. a. ^{1}H-^{15}N HSQC spectra recorded at 800MHz and 25°C of LARP4A NTD in the absence and the presence of oligoA15 RNA (black and red respectively). Severe broadening is observed in the spectrum of the complex. **b.** Zoomed-in regions of the ^{1}H-^{15}N HSQC spectra recorded in the absence and the presence of oligoA15 for the LARP4A NTD (top) and La module in isolation (bottom). Representative La module residues experiencing either chemical shift perturbation (CSP), line broadening and/or signal disappearance in both conditions are shown in the left and centre panels (*e.g.* W238, Y239, S236 and G282). Notably some

signals that undergo modest broadening in the La module in isolation disappear in the spectra of the NTD, consistent with the shorter T2 values of the larger NTD protein and the severe signal-to-noise ratio in the latter conditions (see text and panel **a**.). The right panel shows LARP4A La module resonances that are unaffected in both conditions.

Figure S13

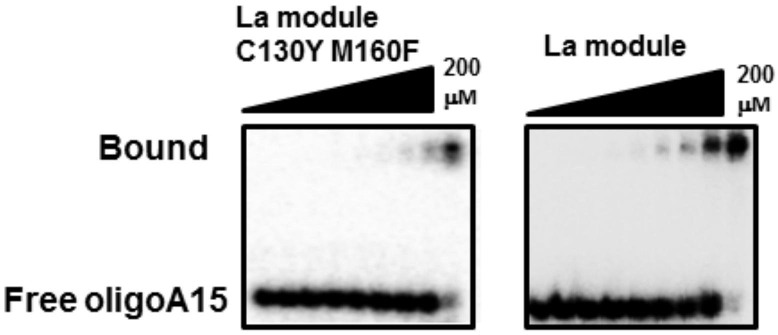

Figure S13. LARP4A mutations within the conserved six residues of the LaM do not affect LARP4A-oligoA interaction. The interaction of LARP4A La module C130YM160F mutant with ^{32}P-oligoA15 was assessed by EMSA. *Left*, C130YM160F double mutant La module and *right* wild type La module. Free and bound oligoA15 are indicated. The experiments were conducted as described in the methods with protein concentrations 0, 1.6, 3.1, 6.3, 12.5, 25, 50, 100 and 200 µM.

References

1. Koradi, R., Billeter, M. and Wuthrich, K. (1996) MOLMOL: a program for display and analysis of macromolecular structures. *Journal of Molecular Graphics*, **14**, 51-55, 29-32.
2. Waterhouse, A.M., Procter, J.B., Martin, D.M., Clamp, M. and Barton, G.J. (2009) Jalview Version 2--a multiple sequence alignment editor and analysis workbench. *Bioinformatics*, **25**, 1189-1191.
3. Dosztanyi, Z., Csizmok, V., Tompa, P. and Simon, I. (2005) IUPred: web server for the prediction of intrinsically unstructured regions of proteins based on estimated energy content. *Bioinformatics*, **21**, 3433-3434.
4. Prilusky, J., Felder, C.E., Zeev-Ben-Mordehai, T., Rydberg, E.H., Man, O., Beckmann, J.S., Silman, I. and Sussman, J.L. (2005) FoldIndex: a simple tool to predict whether a given protein sequence is intrinsically unfolded. *Bioinformatics*, **21**, 3435-3438.
5. Ward, J.J., McGuffin, L.J., Bryson, K., Buxton, B.F. and Jones, D.T. (2004) The DISOPRED server for the prediction of protein disorder. *Bioinformatics*, **20**, 2138-2139.
6. Drozdetskiy, A., Cole, C., Procter, J. and Barton, G.J. (2015) JPred4: a protein secondary structure prediction server. *Nucleic Acids Research*, **43**, W389-394.
7. Yachdav, G., Kloppmann, E., Kajan, L., Hecht, M., Goldberg, T., Hamp, T., Honigschmid, P., Schafferhans, A., Roos, M., Bernhofer, M. *et al.* (2014) PredictProtein--an open resource for online prediction of protein structural and functional features. *Nucleic Acids Research*, **42**, W337-343.
8. Micsonai, A., Wien, F., Bulyaki, E., Kun, J., Moussong, E., Lee, Y.H., Goto, Y., Refregiers, M. and Kardos, J. (2018) BeStSel: a web server for accurate protein secondary structure prediction and fold recognition from the circular dichroism spectra. *Nucleic Acids Research*, **46**, W315-W322.
9. Hwang, T.L., van Zijl, P.C. and Mori, S. (1998) Accurate quantitation of water-amide proton exchange rates using the phase-modulated CLEAN chemical EXchange (CLEANEX-PM) approach with a Fast-HSQC (FHSQC) detection scheme. *Journal of Biomolecular NMR*, **11**, 221-226.
